# Plant Terpenoids in Cardioprotection: An Overview of Their Therapeutic Potential

**DOI:** 10.3390/cimb48050479

**Published:** 2026-05-05

**Authors:** José L. Ríos-López, José Blanco-Salas, Guadalupe Cumplido-Laso, María P. Hortigón-Vinagre

**Affiliations:** 1 Department of Vegetal Biology, Ecology and Earth Science, Faculty of Sciences, University of Extremadura, 06006 Badajoz, Spain; joriosl@alumnos.unex.es; 2 Department of Biochemistry, Molecular Biology and Genetics, Faculty of Sciences, University of Extremadura, 06006 Badajoz, Spain; guadalupecl@unex.es

**Keywords:** cardiomyocyte, cardiopathology, essential oils, health, heart, medicinal plant, terpenoids, treatment

## Abstract

Cardiovascular diseases are the leading cause of morbidity and mortality worldwide, making the search for new therapeutic strategies to prevent or mitigate cardiac damage mandatory. Essential oils, long used in traditional medicine, contain terpenoids as their most prominent constituents, and these molecules have emerged as promising cardioprotective agents. The review compiles 45 articles investigating the effects of plant-derived terpenoids on cardiovascular health. Evidence shows that their therapeutic properties rely on their antioxidant, anti-inflammatory, anti-apoptotic, anti-remodeling, antiarrhythmic, antihypertensive, anti-atherosclerotic, antidiabetic and antimicrobial actions. These effects result from the modulation of molecular pathways altered during cardiovascular diseases, resulting in oxidative stress, inflammation, cell death, fibrosis, ion channel dysregulation, alteration of lipid metabolism and glucose homeostasis. Key mechanisms of terpenes healing properties include activation of endogenous antioxidant defense—mainly via Nrf2-, inhibition of NLRP3 inflammosome-mediated pyroptosis and reduction in lipid oxidation involved in atherosclerotic plaque formation. Their therapeutic potential is reinforced by low toxicity profiles and broad botanical availability. However, challenges related to their translation to therapeutic practice remain unresolved, such as low bioavailability, limited yield and scarce results in human in vitro models. Future research should focus on nano- and micro-delivery systems, biotechnological production strategies and the use of human induced pluripotent stem cell-derived cardiomyocytes. Despite these limitations, terpenes represent valuable templates for developing more potent and clinically viable therapeutic agents. Further studies of this family are encouraged due to its promising ability to treat cardiovascular disorders.

## 1. Introduction

Since ancient times, plants have been widely used in natural medicine. Nowadays, thanks to new technologies, scientists have been able to analyze the different compounds present in these plants and study their molecular mechanisms of action in biological models, making possible their use as active ingredients in numerous drugs [1]. Of all the compounds, essential oils may be among the more important components in terms of healing properties [2]. In their composition, terpenoids are the most abundant group within essential oils, and they are commonly the compounds responsible for their healing properties [3,4]. Terpenoids are a vast family of compounds derived from isoprene. They are secondary metabolites in plants in which they play an important role in plant defense and chemical interaction (e.g., attracting insects to facilitate pollination) [5]. They also participate in many cellular reactions, and, in essential oils, they are responsible for conferring the oils’ physical properties [4].

**Figure 1:**
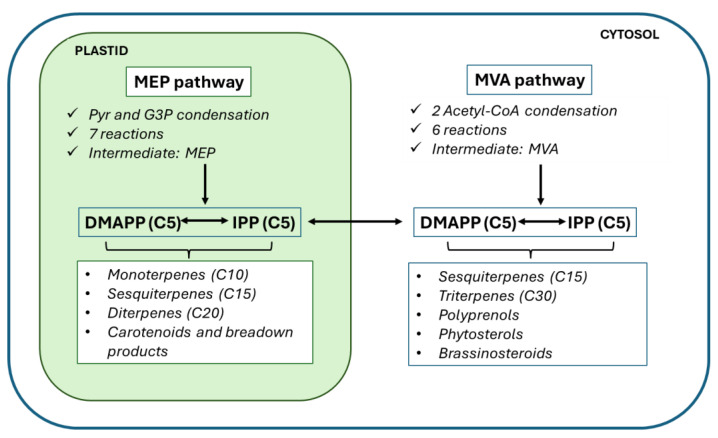
Schematic representation of the terpenoid biosynthetic pathways. Adapted from Pazouki & Niinemets (2016) [9].

Terpenoids are hydrophobic and volatile molecules [6]; they comprise the largest and most diverse class of compounds produced by plants. Their structure is formed by isoprene units, and they are classified according to the number of repeats of isoprene units on the chemical skeleton. Their precursors are two C5 building blocks, isopentenyl diphosphate (IPP) and its isomer dimethylallyl diphosphate (DMAPP) [7]. IPP and DMAPP are produced by two biosynthetic pathways: the mevalonic acid (MVA) pathway, which takes place in the cytosol, and methylerythritol phosphate (MEP) pathway, which is located in the plastid [5]. The presence of two pathways enables their specialization and allows the compartmentalization of isoprenoid pools, although crosstalk between both pathways has been demonstrated in several species, where IPP, DMAPP and C10-15 prenyl diphosphate intermediates can be exchanged between plastids and the cytosol [7]. The biosynthesis of sesquiterpenoids, polyprenols, phytosterols, brassinosteroids and triterpenoids takes place using precursors from the MVA pathway in the cytosol whereas hemi-, mono-, and diterpenoids, carotenoids and their breakdown products, such as cytokinins, gibberellins, tocopherols, plastoquinones and chlorophyll, are produced using C5 precursors of the MEP pathway in plastids [5]. The MVA pathway starts with the condensation of two acetyl-CoA molecules and has six enzymatic reactions to form IPP and DMAPP whereas the MEP pathway comprises seven enzymatic steps, starting with the condensation of pyruvate and glyceraldehyde-3-phosphate (GAP) (Figure 1) [5]. It is estimated that there are hundreds of genes responsible for encoding the different enzymes involved in terpene formation [8].

Cardiomyocytes are special muscle cells present in the heart, where they constitute 49.2% of the cell mass and give this organ its most notable characteristic, the contractility [10,11,12]. The highest specialization of these cells leads to a partial loss of their proliferative capacity, which is responsible for the inability to recover lost cardiomyocytes. Therefore, in the event of cell damage and death, the workload is shared among the remaining cardiomyocytes, thus increasing their workload. The constant loss of cells and the increased workload of the remaining ones are the origin of many heart diseases [10,12]. In addition, there are some pathologies that accelerate this process and cause heart diseases at an early age, including insulin resistance, oxidative stress or problems in embryonic development [13,14]. Because heart diseases cause the highest rate of morbidity and mortality worldwide, significant efforts are being made to better understand the molecular mechanisms underlying them and to develop therapeutics approaches aimed at preventing and treating them [11].

The need to maintain cardiovascular health and the rise in new studies demonstrating novel medicinal properties in plant-derived compounds has prompted research into articles that have focused their studies on botanical substances with cardioprotective properties. Thus, it has been shown that many compounds present in essential oils, such as terpenoids, possess properties to treat or prevent many heart diseases. It has also been found that some of these compounds have multiple properties due to their ability to target different heart diseases or to address the same heart disease from different pathways [15,16,17]. All these properties have led to a deep study of this family in order to search for potential medications to treat cardiovascular diseases. For example, one of these studies demonstrates new properties of Andalusian thyme oil (*Thymbra capitata*) and its terpenes applied to animal cells, in this case neonatal rat cardiomyocytes, exposing the potential for its use in pharmacology [18]. This species has been traditionally used as a condiment and preservative [19]. Medicinally, it has been used for disorders of the genitourinary system [20], respiratory system [21], and also for diseases of the skin, muscles, and bones [22]. Given this background, a study proposed evaluating its antioxidant activity [23], and the results encouraged Hortigón-Vinagre et al. [18] to use its essential oil against heart conditions.

In light of this background, the objective of this work is to carry out a literature review compiling scientific works showing the cardioprotective role of terpenoids.

To conduct this review, a comprehensive literature search was performed focusing on studies demonstrating the cardioprotective effect of plant-derived terpenoids. Articles evaluating the use of essential oils containing terpenoids in their composition as well as studies assessing isolated terpenoids as therapeutic agents for heart disease were included. The search was carried out using the following keywords: cardiomyocyte; cardiopathology; essential oils; health; heart; medicinal plant; terpenoids; terpenes; treatment. Most of the selected articles were published within the last 20 years, with particular emphasis placed on more recent studies. The databases consulted were Elsevier, PubMed, ScienceDirect, Web of Science, Scielo, and Google Scholar. The management and organization of bibliographic sources was carried out using the reference management software “Mendeley” (www.mendeley.com).

## 2. Results and Discussion

A total of 45 articles were obtained and analyzed. Each article generally addresses one or more biological properties of terpenoids, so they were classified according to the biological properties related to heart disease. It should be noted that some studies are repeated between sections, as some articles describe multiple properties for the same compound.

**Figure 2:**
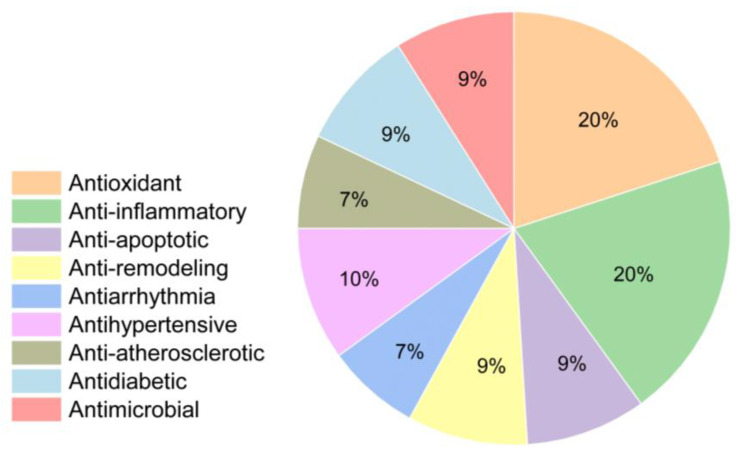
Distribution of articles according to their biological effect.

Of the total articles analyzed, 28 showed antioxidant effects, 28 anti-inflammatory effects, and 12 anti-apoptotic effects. These three effects are grouped together in the section dedicated to the protective effect against cell death-related mechanisms. In addition, there are 12 publications on anti-remodeling effects, 10 on anti-arrhythmia effects, 13 on antihypertensive effects, 10 on anti-atherosclerosis effects, 12 on antidiabetic effects, and 12 on antimicrobial effects (Figure 2).

### 2.1. Protective Effect Against Cell Death

This section groups together antioxidant, anti-inflammatory, and anti-apoptotic effects, since all three confer protection against cell death. Additionally, compounds able to keep cells alive and healthy are included, since cardiomyocyte death would trigger a greater workload for the rest of the heart tissue, causing greater stress, poorer function and eventually the development of heart failure [10,12,14].

Tissue loss is mainly due to three principal mechanisms: apoptosis, necrosis, and pyroptosis. Apoptosis is also known as programmed cell death since it is triggered by certain signaling pathways to maintain and protect tissue; necrosis is the destruction of cells due to the loss of integrity of the cell membrane; and pyroptosis is a type of cell death triggered by inflammatory molecules such as caspase-1 which cleave the protein gasdermin D. The N-terminal fragment of gasdermin-D forms pores in the cell membrane through which inflammatory molecules are released [24,25].

**Figure 3:**
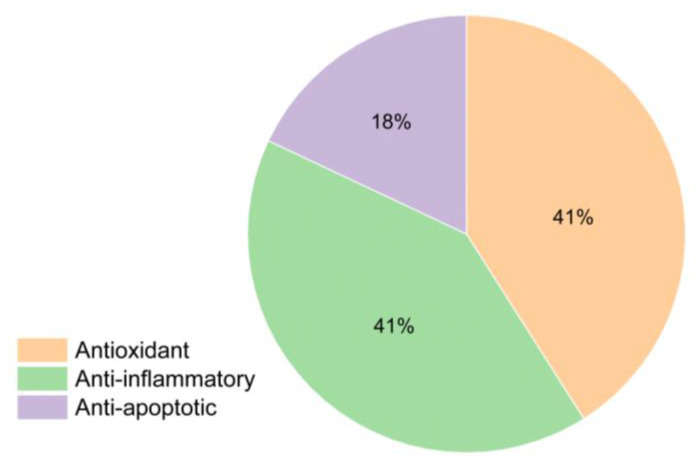
Division of articles focused on compounds with ability to prevent cardiomyocyte death.

A total of 40 articles were identified that include terpenoids with the ability to prevent cardiomyocyte death. Of these articles, 28 deal with death caused by oxidative stress, 28 with death caused by inflammation or pyroptosis, and 12 with apoptosis (Figure 3).

#### 2.1.1. Antioxidant Effect

This section includes results demonstrating the antioxidant properties of terpenoids and their molecular mechanisms to tackle oxidative stress. A total of 28 articles were identified on this topic (Table 1).

Oxidative stress is one of the most prominent factors when it comes to heart disease. This occurs when reactive oxygen species (ROS) overwhelm the cells’ antioxidant defenses [26]. The high workload of cardiomyocytes to keep the heart functioning implies high energy requirements to maintain the ion gradient and the contractility machinery, and most of the energy is obtained from oxidative phosphorylation [27]. Of all the compounds generated in the respiratory chain, NADPH plays an important role in the generation of ROS through NADHP oxidase activity. These reactive species, if not eliminated, cause problems throughout the cell due to their ability to react with proteins, lipids and DNA, which causes oxidative damage to these molecules and alters their function [28]. The increase in oxidative species and damage to macromolecules triggers apoptosis in the cell by activating caspases [25].

The antioxidative capacity (AC) of botanicals is well known [18,29]. Terpenoids are lipophilic molecules with no aromatic ring; their AC relies on the large number of conjugated double bonds in their chemical structure, which provide the ability to stabilize the donated electrons [30]. It has also been shown that some compounds can activate natural cellular defenses against oxidative compounds by enabling them to stimulate the formation of enzymes and antioxidant compounds such as superoxide dismutase (SOD) and catalase (CAT), glutathione peroxidase (GPx), and glutathione S-transferase (GST) as well as reduced glutathione (GSH) [31,32,33,34,35,36]. This effect on antioxidant defense has been demonstrated in the methanolic extract of *Sansevieria roxburghiana*, rich in the triterpene lupeol [37], whose antioxidant properties and cardioprotective action have been shown against cyclophosphamide-induced cardiotoxicity [38].

The expression of antioxidant enzymes is regulated by the Nrf2 pathway, as demonstrated in a study showing the cardioprotective effect of myrrh essential oil, which is rich in sesquiterpenes, where Nrf2 expression is upregulated by 6–9-fold, compared with ISO-induced MI, when threatened with ISO and myhr essential oil [39]. A similar effect has been observed with triterpenes ursolic, maslinic, betulonic and oleanoic acids, all of them with proven antioxidant properties due to their ability to reduce oxidative stress by activating the Nrf2 pathway and thereby increasing antioxidant enzymes (CAT, SOD, GPx) [31,32,33,34,40,41,42,43,44,45,46,47]; this could explain the antioxidant effect of plants such as *Senna auriculata*, *Liquidambar orientalis* and *Vaccinium macrocarpon.* The triterpene betulin also promotes Nrf2 nuclear translocation, which is explained by the activation of the AMPK/Nrf2 pathway through enhanced interaction between AMPK and Nrf2 [48].

The antioxidant properties of essential oil-derived terpenes also rely on their ability to preserve healthy mitochondria, mitigating the extent of oxidative stress. In this way, the triterpene ursolic acid acts, improving mitochondrial function through DRP1 (Dynamic Related Protein) inhibition [49]. A similar effect was achieved by the triterpene betulinic acid, in this case through the upregulation of PINK1/Parking involved in activating mitophagy to remove dysfunctional mitochondria responsible for ROS production [50]. The effect of betulin on the AMPK/Nrf2 signaling axis also resulted in the alleviation of mitochondrial dysfunction [48].

Besides the most common mechanism to reduce oxidative stress, which is through activation of the Nrf2 pathway, Zhan et al. described the ability of the triterpene lupeol to prevent oxidative stress through inhibition of the BMP4/NOX1 pathway. BMP-4 (Bone Morphogenetic Protein) can activate NOX1 (NADPH oxidase 1) and upregulate COX-2 (Ciclooxygenase-2), enhancing ROS burst in cardiovascular synthesis [51].

**Table 1:** Studies that demonstrate the antioxidant effect.

Plant	Terpenoids	Study	Effect	Reference
*Ocimum basilicum* (Basil)	Linalool, eucalyptol, 3,7-dimethyl-1,3,6-octatriene, β-pinene	In vitro, in vivo	↓ ROS	[27]
*Juniperus phoenicea* (Sabina negral), *Laurus nobilis* (Laurel), *Melaleuca armillaris* (Bracelet honey myrtle), *Thymbra capitata* (Andalusian thyme)	*α*-Pinene, *β*-myrcene, *p*-cymene, *γ*-terpinene, *β*-caryophyllene, *β*-elemene, *α*-himachalene, *γ*-muurolene, *β*-caryophyllene oxide, thymol	In vitro, in vivo	↓ ROS	[52]
*Coriander sativum* (Coriander), *Apium graveolens* (Celery), *Ocimum minimum* (Bush-basil)	Linalool, *α*-pinene, camphor, *p*-cymene	In vitro	↓ ROS	[53]
*Thymbra capitata* (Andalusian thyme)	Carvacrol, *p*-cymene, *γ*-terpinene, *α*-terpinene, *α*-thujene, *α*-pinene, camphene, myrcene, linalool, (E)-caryophyllene	In vitro, in vivo	↓ ROS	[18]
*Nardostachys jatamansi* (Nard)	Calarene, *β*-maaliene, 9-aristolene	In vitro	↓ ROS + antioxidant enzymes activation	[28]
*Lavandula* spp., *Salvia rosmarinus* (Rosemary), *Salvia officinalis* (Sage)	Camphorquinone	In vivo	↓ ROS + response activation against ROS	[54]
*Liquidambar orientalis* (Oriental sweetgum)	Oleanolic acid, betulinic acid, corosolic acid, maslinic acid, epibetulinic acid, betulonic acid	In vitro, in vivo	↓ ROS + antioxidant enzymes activation (SOD)	[55]
*Senna auriculata* (Avaram senna)	Oleanolic acid	In vitro, In vivo	↓ ROS + antioxidant enzymes activation	[56]
*Ceriops decandra* (Clumped yellow mangrove)	Isosteviol	In vivo	↓ ROS + mitochondrial membrane potential maintenance	[17]
*Nigella sativa* (Black cumin)	Thymoquinone, carvacrol, 4-terpineol, *α*-pinene, thymol, t-anethole, thymohydroquinone, ithymoquinone, *p*-cymene	In vitro, in vivo	↓ ROS + antioxidant enzymes activation (SOD, CAT)	[57]
*Scrophularia ningpoensis* (Ningpo figwort)	Sugiol, lupeol, ursolic acid, oleanonic acid, eucalyptolic acid, scrokoelziside A, scrokoelziside B, 14-deoxyandrographolide	In vivo	↓ ROS	[58]
*Melissa officinalis* (Lemon balm)	Citronellal, thymol, citral, *β*-caryophyllene, caryophyllene oxide, limonene, germacrene, ursolic acid, oleanolic acid	In vivo	↓ ROS	[59]
*Bougainvillea glabra* (Lesser bouginvillea)	Oleananoic acid acetate, oleoside dimethyl ester, goyaglucoside, oleanolic acid 3-O-beta-D-glucosiduronic acid, phytol, squalene, geranylgeraniol	In vitro	↓ ROS	[60]
*Sequoia sempervirens* (Coast redwood)	*α*-Pinene, *β*-pinene, myrcene, limonene	In vivo	↓ ROS + antioxidant defenses activation (SOD, CAT, GPx, GSH)	[31,36]
*Vaccinium macrocarpon* (Large cranberry)	Ursolic acid	In vivo	↓ ROS	[61]
*Matricaria chamomilla* (Chamomille)	α-Bisabolol	In vitro, in vivo	↓ ROS + antioxidant enzymes activation (SOD, CAT)	[26]
*Aconitum carmichaelii* (Chinese aconite), *Atractylodis macrocephalae* (Báizhú), *Paeoniae alba* (Bai Shao), *Panax ginseng* (Asian ginseng), *Salvia miltiorrhiza* (Red sage), *Wolfiporia extensa* (Hoelen), *Zingiber officinale* (Ginger)	Linalool, *α*-pinene, camphor, *p*-cymene	In vitro	↓ ROS	[62]
*Pyrola* spp. (*pyrolae herba*)	*β*-sitosterol, ursolic acid, uvaol, 3-hydroxy-11-oxo-oleanolic acid, 3,11-dioxo-oleanolic acid, monotropin, pisumionoside, daucosterol, pomolic acid, oleonolic acid, maslinic acid, collosic acid, taraxerol, miricadiol, betulin, ziyuglucoside I	In vitro	↓ ROS	[63]
*Salvia miltiorrhiza* (Red sage)	Miltrione	In vivo	↓ ROS	[62]
*Eleutherococcus* spp.	Eleutherococcus lupane triterpenes	In vitro, in vivo	↓ ROS + antioxidant enzymes activation (SOD, CAT)	[64]
*Amaryllidaceae*, *Asparagaceae*, *Asteraceae*, *Dioscoreaceae*, *Fabaceae*, *Liliaceae*, *Plantaginaceae*, *Smilacaceae*, *Solanaceae*, *Zygophyllaceae* families	Triterpenoid glycosides derived saponins	In vivo	↓ ROS generation	[65]
*Astragalus* spp., *Ginkgo biloba* (Ginkgo), *Panax pseudoginseng* (Notoginseng), *Phyllanthus emblica* (Emblic)	Astragaloside IV, diosgenin, ginsenoside Re, lupeol, oleanolic acid, phylloemblycin B, 20(S)-protopanaxtriol, ursolic acid	In vivo	↓ ROS	[66]
*Artemisia annua* (Sweet wormwood), *Betula* spp., *Gardenia jasminoides* (Gardenia), *Hypopterygium* gns., *Lonicera japonica* (Japanese honeysuckle), *Panax ginseng* (Asian ginseng), *Rabdosia rubescens* (Donglingcao), *Tripterygium wilfordii* (Thunder duke vine)	Artemisinin, betulin, celastrol, dioscina, geniposide, ginsenoside Rg3, oridonin, sweroside, triptolide	In vivo	↓ ROS	[24]
*Aralia* spp.	Elatoside F, araloside C, chikusetsusaponin IVa, chikusetsusaponin IVa, elatoside I, oleanolic acid 3-O-*β*-D-glucopyranosyl(1 → 3)-*α*-L-rhamnopyranosyl (1 → 2)-*α*-L-arabinopyranoside, kaurenoic acid, continental acid, 7-oxo-ent-pimara-8 (14), 15-dien-19-oic acid, 3-O-{*β*-D-glucopyranosyl-(1 → 2)-[*β*-D-glucopyranosyl-(1 → 3)]-*β*-D-glucuronpyranosyl} 28-O-*β*-D-glucopyranosyl ester of oleanolic acid, 3-O-{*β*-D-glucopyranosyl-(1 → 2)-[*β*-D-glucopyranosyl-(1 → 3)]-*β*-D-glucuronpyranosyl}olean-11,13(18)-diene-28-oic ester 28-O-*β*-D-glucopyranosyl	In vivo	↓ ROS	[67]
*Isodon rubescens* (Donglingcao)	Oridonin, ponicidin, lushanrubescensin H, lushanrubescensin J, rhabdosin A, isodocarpine, rhabdoternin F, shikokianin, lasiodin, parvifolin AA, lasiodonin, lasiodoninacetonide, rostorin, isojiangrubesin C, isojiangrubesin E, rhabdoternin E, jaridonina, 14- O-acetyl-oridonin, isodonoiol, isodonal, rhabdosin B, efusanin A, xerophinoid B and 7,14-O-(1-methylethylidene) oridonin, ursolic acid, oleanic acid, *β*-sitosterol, *α*-amyrin, daucosterol, betulin, eryhrodiol, stigmasterol	In vivo	↓ ROS	[68]
*Cucurbitaceae* family	Cucurbitacin triterpenoids	In vitro, in vivo	↓ ROS	[69]
*Falcaria vulgaris* (Sickleweed)	*α*-Pinene, spathulenol, carvacrol, limonene	In vitro, in vivo	↓ ROS + antioxidant enzymes activation (SOD, CAT)	[70]
*Urtica dioica* (Nettle)	4,7-Megastigma-diene-3, 9-diol; (3S,6R,7E,9R)-form, 3-ketone, 9-O-[b-D-glucopyranosyl-(1 ⟶ 2)-*β*-d-glucopyranoside], 1-(3, 4-dihydroxyphenyl)-1, 2-propanediol; 3′-Me ether, (9Z,11E)-1, 3-hydroxy-9, 11-octadeca-dienoic acid, hexahydrofarnesyl acetone, geranyl acetone, (E)-anethole, *p*-hydroxybenzaldehyde, b-ionone	In vitro, in vivo	↓ ROS + antioxidant enzymes activation (SOD, GHS)	[71]
*Commiphora myrrha*	*β*-Elemene, *δ*-elemene, *β*-bourbonene, *α*-bergamotene, germacrene A and B, furanoeudesma-1,3-diene, lindestren, curzerene	In vivo	↑ Nrf2 + antioxidant defenses activation (SOD, CAT, GSH)	[39]
*Lagerstroemia speciosa* L.	Corosolic acid, ursolic acid	In vivo	↑ Nrf2 + antioxidant defenses activation (SOD, CAT, GSH, GPx) + ↓ MDA	[47]
*Sansevieria roxburghiana*	Lupeol	In vivo	antioxidant defenses activation (SOD, GSH) + ↓ MDA	[37]

↓ reduce; ↑ activate.

Besides the pharmacological properties of terpenoids, scientists can modify their chemical structure to create synthetic derivatives with even more pronounced pharmacological action. Such is the case of isosteviol, a diterpenoid with well-known cardioprotective properties which has been used as a starting point to create synthetic derivatives with stronger healing properties. Among the 47 isosteviol derivatives tested in zebrafish, one of them showed the best efficacy to prevent cardiac damage and dysfunction. The molecular mechanism of this new chemical entity underlies its ability to prevent ROS accumulation [17].

#### 2.1.2. Anti-Inflammatory Effect

This section addresses the anti-inflammatory effects of plant terpenes mainly due to their ability to interact with certain inflammatory pathways. A total of 27 articles related to this action were found (Table 2).

Inflammation is a response of the immune system, triggered when it detects certain self or foreign molecules [72]. In the case of pathogens, components of the bacterial cell wall act as triggers for this response [72]. The binding of these molecules to macrophages induces the release of pro-inflammatory mediators, which initiate the inflammatory process in the area. Inflammation is characterized by increased permeability of local blood vessels, allowing the influx of immune cells and soluble mediators [72]. In short-term responses, inflammation contributes to minimizing tissue damage. However, in prolonged inflammatory responses, the opposite effect can occur, leading to necrosis in the affected area due to an overactive immune response and the accumulation of ROS [72].

The anti-inflammatory effect is primarily achieved by interrupting this response, thereby preventing prolonged inflammation that can lead to tissue damage. In the context of heart disease, NLRP3 inflammasomes play a central role in mediating cardiac tissue injury. Inflammasomes are multiprotein complexes formed by NLRP3, ASC, and Pro-caspase-1, which amplify the inflammatory response and trigger pyroptosis, an atypical form of cell death. This complex is assembled when there is a high flow of potassium ions and ROS [24].

Another problem related to inflammation is myocarditis, which is inflammation of the cardiac muscle tissue. This inflammation can be associated with pathogens, certain types of drugs, or with an autoimmune reaction. Prolonged myocarditis can lead to arrhythmias, heart failure, and even death [66]. Currently, medications for myocarditis are not as effective as expected, but some studies have shown that some natural products may be good therapeutic agents in combination with conventional drugs [66].

It should be noted that the antioxidant and anti-inflammatory effects are closely linked, since one of the causes of inflammation is the increase in ROS [57].

Within the molecular mechanisms related to anti-inflammatory effects, terpenoids can interact with different molecules such as interleukin 1 (IL-1), interleukin 1*β* (IL-1*β*), interleukin 6 (IL-6), interleukin 10 (IL-10), tumor necrosis factor *α* (TNF-*α*), nitric oxide synthase (iNOS), prostaglandin-endoperoxide synthase (COX2), nitric oxide (NO), interleukin 8 (IL-8), interferon *ɣ* (IFN-*ɣ*), interleukin 12 (IL-12) and dinoprostone (PGE2) [26,57,58,67,73]. After compiling the articles, it is worth highlighting the properties of *Matricaria chamomilla* and *Scrophularia ningpoensis*. *α*-bisabolol, firstly obtained from *Matricaria chamomilla*, a plant popularly used in traditional medicine for its healing properties, has shown promising results in ameliorating myocardial infarction (MI) damage due to its ability to attenuate oxidative stress and inflammation by inhibiting the NLRP3 inflammasome activation and TLR4-NF*κ*B/MAPK signaling pathways, thereby reducing pyroptosis. In addition, α-bisabolol protects against *β*-adrenergic agonist-induced myocardial infarction in rats by attenuating inflammation, lysosomal dysfunction, NLRP3 inflammasome activation and modulating autophagic flux [26,74].

*Scrophularia ningpoensis* resembles the actions of *Swertia chirayita* but has the added effect of reducing NF-*κ*B molecules. The anti-inflammatory power of *Scrophularia ningpoensis* extracts could be due to the anti-inflammatory activity of iridoid glycosides such as harpagoside, scropolioside A [58] and the triterpene ursolic acid, which has been shown to downregulate the NF*κ*B pathway, a property also described for the triterpene betulinic acid [31,44]. Another triterpene with proven anti-inflammatory properties is corosolic acid; it exerts its anti-inflammatory properties through activation of peroxisome proliferator-activated receptor gamma (PPAR*γ*), a nuclear receptor capable of inhibiting the interaction of NF*κ*B with DNA, preventing the inflammatory response triggered by NF*κ*B [75]. The ability of corosolic acid to activate PPAR is shared by other triterpenes such as lupeol, a compound with anti-inflammatory properties due to its ability to inhibit NF*κ*B [37,76].

**Table 2:** Studies demonstrating the anti-inflammatory effect.

Plant	Terpenoids	Study	Effect	Reference
*Lavandula* spp., *Salvia rosmarinus* (Rosemary), *Salvia officinalis* (Sage)	Camphorquinone	In vivo	↓ Inflammatory marker expression (IL1*α*, IL1*β*, IL6)	[54]
*Liquidambar orientalis* (Oriental sweetgum)	Oleanolic acid, betulinic acid, corosolic acid, maslinic acid, epibetulinic acid, betulonic acid	In vitro, in vivo	↓ Inflammatory marker expression (TNF-*α*, IL-1β)	[55]
*Trypterygiun wilfordii* (Thunder duke vine), *Salvia milthiorrhiza* (Red sage)	Celastrol, cryptotanshinone, geraniol, lycopene, oleanolic acid, thymoquinone, ursolic acid	In vitro, in vivo	↓ Inflammatory markers (IL-6) + ↓ ROS	[77]
*Andrographis paniculata* (Creat)	Andrographatoside, oleanolic acid, andrographolide, 3-O-*β*-D-glucopyranosilandrographolide, 3-Oxo-14-deoxy-11,12-didehydroandrographolide, neoandrographolide, 14-deoxyandrographolide, andrograpanin, 3-O-*β*-D-glucosyl-14 -deoxyandrographolide, 6′-acetylneoandrographolide, 14-deoxy-17-*β*-hydroxyandrographolide, 19-O-[*β*-D-apiofuranosyl(1 → 2)-*β*-D-glucopyranoyl]-3,14 dideoxyandrographolide, isoandrographolide, 14-deoxy-11-oxo-andrographolide, 14-deoxy-12-hydroxyandrographolide, 8,17-epoxy-14-deoxyandrographolide, 3-O-*β*-D-glucosyl-14-deoxyandrographolide, 12S-hydroxyandrographolide, 14-Deoxy-15-isopropylidene-11,12-didehydroandrographolide, bisandrographolide A, bisandrographolide B, bisandrographolide C, bisandrographolide D, bisandrographolide E, bisandrographolide F, bisandrographolide G, bisandrographolide ether	In vivo	↓ Inflammatory marker expression (NF-*κ*B, TNF-*α*)	[78]
*Isodon rubescens* (Donglingcao)	Oridonin	In vitro, in vivo	Macrophages regulation +↓ inflammatory response (Nrf2, NF-*κ*B)	[79]
*Nigella sativa* (Black cumin)	Thymoquinone, carvacrol, 4-terpineol, *α*-pinene, thymol, *t*-anethole, thymohydroquinone, ithymoquinone, *p*-cymene	In vitro, in vivo	↓ Inflammatory markers (IL-6, IL-1*β*, TNF-*α*, iNOS, COX2)	[57]
*Scrophularia ningpoensis* (Ningpo figwort)	Sugiol, lupeol, ursolic acid, oleanonic acid, eucalyptolic acid, scrokoelziside A, scrokoelziside B, 14-deoxy andrographolide	In vivo	↓ Inflammatory markers (IL6, IL-1*β*, TNF-*α*) + ↑Inflammatory marker (IL10) + NF-*к*B inhibition	[58]
*Bougainvillea glabra* (Lesser bouginvillea)	Oleananoic acid acetate, oleoside dimethyl ester, goyaglucoside, oleanolic acid 3-O-beta-D-glucosiduronic acid, phytol, squalene, geranylgeraniol	In vitro	↓ Inflammation	[60]
*Sequoia sempervirens* (Coast redwood)	*α*-Pinene, *β*-pinene, myrcene, limonene	In vivo	↓ Inflammatory markers (TNF-*α*, IL-6, NF-*k*B)	[36,80]
*Vaccinium macrocarpon* (Large cranberry)	Ursolic acid	In vivo	↓ Inflammation	[61]
*Matricaria chamomilla* (Chamomille)	*α*-Bisabolol	In vitro, in vivo	↓ Inflammatory markers (IL-1*β*, IL-6, TNF-*α*) + ↓ NLRP3 complex	[26,74]
*Houttuynia cordata* (Fish mint)	Houttuynin, decanal, trans-caryophyllene, decanoic acid, camphene, *β*-pinene, lauraldehyde, *α*-pinene, limonene, nonanol and linaloolbornyl acetate, methyl n-nonyl ketones, beta myrcene, monoterpene, 4-terpineol, caryophyllene oxide, derivatives phenylpropene, sesquiterpenes, oxidized diterpenes	In vivo	↓ Inflammatory markers (TNF-*α*, IL-6) + ↓Sirt-1 expression	[81]
*Salvia miltiorrhiza* (Red sage)	Miltrione	In vivo	↓ Inflammation	[62]
*Eleutherococcus* spp.	Eleutherococcus lupane triterpenes	In vitro, in vivo	↓ Inflammatory markers (TNF-*α*, IL-1)	[64]
*Amaranthus hybridus* (Green amaranth), *Barringtonia acutangular* (Freshwater mangrove)	Protopanaxatriol, cucurbitacin B	In vitro, in vivo	Immunological molecules regulation (IL-10, IFN-*ɣ*)	[82]
*Sophora flavescens* (Shrubby sophora)	Kuraridine, L-maackiain, kushenin, *β*-sitosterol, lupenone, *β*-amyrin, lupeol, poncimarin	In vitro	↓ Inflammatory markers (COX-2, iNOS, NO, IL-8, IL-6, TNF-*α*)	[73]
*Amaryllidaceae*, *Asparagaceae*, *Asteraceae*, *Dioscoreaceae*, *Fabaceae*, *Liliaceae*, *Plantaginaceae*, *Smilacaceae*, *Solanaceae*, *Zygophyllaceae* families	Triterpenoid glycosides derived saponins	In vivo	↓ Inflammatory markers (IL, TNF)	[65]
*Astragalus* spp., *Ginkgo biloba* (Ginkgo), *Panax pseudoginseng* (Notoginseng), *Phyllanthus emblica* (Emblic)	Astragaloside IV, diosgenin, ginsenoside Re, lupeol, oleanolic acid, phylloemblycin B, 20(S)-protopanaxtriol, ursolic acid	In vivo	↓ Inflammatory markers (TNF-*α*, IL-1*β*, IL-6, IKKβ, I*κ*B*α*, p65, NF-*κ*B, MCP-1)	[66]
*Artemisia annua* (Sweet wormwood), *Betula* spp., *Gardenia jasminoides* (Gardenia), *Hypopterygium* gns., *Lonicera japonica* (Japanese honeysuckle), *Panax ginseng* (Asian ginseng), *Rabdosia rubescens* (Donglingcao), *Tripterygium wilfordii* (Thunder duke vine)	Artemisinin, betulin, celastrol, dioscina, geniposide, ginsenoside Rg3, oridonin, sweroside, triptolide	In vivo	Inflammatory markers modulate (TNF-*α*, NF-*κ*B, IL-1*β*) + ↓ NLRP3 complex	[24]
*Aralia* spp.	Elatoside F, araloside C, chikusetsusaponin IVa, chikusetsusaponin IVa, elatoside I, oleanolic acid 3-O-*β*-D-glucopyranosyl(1 →3)-*α*-L-rhamnopyranosyl (1 → 2)-*α*-L-arabinopyranoside, kaurenoic acid, continental acid, 7-oxo-ent-pimara-8 (14), 15-dien-19-oic acid, 3-O-{*β*-D-glucopyranosyl-(1 → 2)-[*β*-D-glucopyranosyl -(1 → 3)]-*β*-D-glucuronpyranosyl} 28-O-*β*-D-glucopyranosyl ester of oleanolic acid, 3-O-{*β*-D-glucopyranosyl-(1 → 2)-[*β*-D-glucopyranosyl -(1 → 3)]-*β*-D-glucuronpyranosyl}olean-11,13(18)-diene-28- oic ester 28-O-*β*-D-glucopyranosyl	In vivo	↓ Inflammatory markers (NO, PGE_2_, TNF-*α*, IL-1*β*) + ↓ NLRP3 complex	[67]
*Cucurbitaceae* family	Cucurbitacin triterpenoids	In vitro, in vivo	↓ IFN-*γ* + ↑ IL-10	[69]
*Isodon rubescens* (Donglingcao)	Oridonin, ponicidin, lushanrubescensin H, lushanrubescensin J, rhabdosin A, isodocarpine, rhabdoternin F, shikokianin, lasiodin, parvifolin AA, lasiodonin, lasiodoninacetonide, rostorin, isojiangrubesin C, isojiangrubesin E, rhabdoternin E, jaridonina, 14-O-acetyl-oridonin, isodonoiol, isodonal, rhabdosin B, efusanin A, xerophinoid B and 7,14-O-(1-methylethylidene) oridonin, ursolic acid, oleanic acid, *β*-sitosterol, *α*-amyrin, daucosterol, betulin, eryhrodiol, stigmasterol	In vivo	↓ Inflammatory markers (NF-*κ*B, TNF-*α*, L-1*β*, IL-6)	[68]
*Falcaria vulgaris* (Sickleweed)	*α*-Pinene, spathulenol, carvacrol, limonene	In vitro, in vivo	↓ Inflammatory cells	[70]
*Panax notoginseng* (Chinese ginseng)	*Panax notoginseng* saponins	In vivo	↓ Inflammation	[83]
*Urtica dioica* (Nettle)	4,7-Megastigma-diene-3,9-diol; (3S,6R,7E,9R)-form, 3-ketone, 9-O-[b-D-glucopyranosyl-(1 ⟶ 2)-b-d-glucopyranoside], 1-(3,4-dihydroxyphenyl)-1,2-propanediol; 3′-Me ether, (9Z,11E)-1,3-hydroxy-9,11-octadeca-dienoic acid, hexahydrofarnesyl acetone, geranyl acetone, (E)-anethole, *p*-hydroxybenzaldehyde, *β*-ionone	In vitro, in vivo	↓ Inflammatory markers (TNF-*α*, NF-k*β*, IL-1*β*)	[71]
*Artemisia annua* (Sweet wormwood), *Centella asiatica* (Indian pennywort), *Ginkgo biloba* (Ginkgo), *Lamiaceae* family, *Ligustrum lucidum* (Chinese privet), *Olea europaea* (Olive), *Swertia mussotii* (Zangyinchen)	Asiatic acid, oleanolic acid, ursolic acid, artemisinin, ginkgolide B	In vitro, in vivo	NF*κ*B path inhibition + NLRP3 and PERK protein inhibition, ↓ Inflammatory markers (TNF-*α*, IL-6, MCP-1, TGF-*β*1) + anti-inflammatory molecules stimulation (GMCSF, IL-10, IL-12)	[84]
*Polygonatum sibiricum* (Siberian Solomon’s Seal)	Curcumenol, geniposide	In vitro, in vivo	NF-*κ*B and p38 MAPK path suppress + ↓ Inflammatory markers (TNF-*α*, IL-1, IL-2, IL-6, IL-17, JNK, ERK) + ↑ Anti-inflammatory markers (IL-4, TGF-*β*1, IL-10, TLR4), ↓ COX-2, iNOS, MLCK	[85]
*Bauhinia* spp.	23-hydroxy-3α-[O-*α*-L-1C4-rhamnopyranosyl-(1″→4′)-O-*α*-L-4C1-arabinopyranosyl-oxy]olean-12-en-28-oic acid O-*α*-L-1C4-rhamnopyranosyl-(1⁗′ → 4⁗)-O-*β*-D-4C1-glucopyranosyl-(1⁗→6‴)-O-*β*-D-4C1-glucopyranosyl ester, champine A	In vivo, in vitro	Edema reduction from 100% to 39,6% + NF-*κ*B inhibition + ↓ inflammatory markers (TNF-*α*, IL-6, IL-8) + ↑ Anti-inflammatory markers (IL-10)	[86]

↓ reduce; ↑ activate.

#### 2.1.3. Anti-Apoptotic Effect

This section presents the results regarding anti-apoptotic effects aside from the causes exposed in Section 2.1.1 and Section 2.1.2, where plants with antioxidant and anti-inflammatory properties were considered since both are the main causes triggering apoptosis. A total of nine articles were found (Table 3).

One way to prevent apoptosis is by targeting the JNK and p38 MAPK signaling pathways. These pathways connect membrane receptors to the nucleus and induce apoptosis in response to extracellular signals, such as inflammatory molecules [16]. Another strategy involves modulating the Bcl-2 and Bax family proteins. Bcl-2 is an anti-apoptotic protein that inhibits Bax, which, when active, triggers mitochondrial apoptosis [55]. Additionally, several studies have demonstrated that molecules affecting the PI3K/Akt signaling pathway can also prevent apoptosis [55].

Since all the anti-apoptotic properties are summarized in Table 3, the anti-apoptotic effect of storax, a resin obtained from *Liquidambar orientalis* and widely used in Eastern folk medicine, can be highlighted for its ability to prevent cardiomyocyte apoptosis by reducing the Bax/Bcl-2 ratio. Further studies have suggested the PI3K/Akt pathway as storax target [55]. A similar effect on Bax and Bcl-2 proteins was observed using *Polygonatum sibiricum* polysaccharide on acute HF rats [87].

Several triterpenes have shown anti-apoptotic effects; they include ursolic, oleanoic, maslinic and betulinic acid. This effect is due to the restoration of Bcl-2 levels and decrease in the pro-apoptotic proteins Bax and caspase 3 [31,40,43,44,45,46,49]. Since most of these triterpenes have been described in the composition of storax, this could explain its anti-apoptotic activity.

**Table 3:** Studies that demonstrate the anti-apoptotic effect.

Plant	Terpenoids	Study	Effect	Reference
*Origanum vulgare* (Oregano), *Thymus vulgaris* (Common thyme)	Carvacrol	In vitro, in vivo	ERK action inhibition	[16]
*Liquidambar orientalis* (Oriental sweetgum)	Oleanolic acid, betulinic acid, corosolic acid, maslinic acid, epibetulinic acid and betulonic acid	In vitro, in vivo	↓ Bax/Bcl-2 ratio + Regulates PI3K/Akt pathway	[55]
*Scrophularia ningpoensis* (Ningpo figwort)	Sugiol, lupeol, ursolic acid, oleanonic acid, eucalyptolic acid, scrokoelziside A, scrokoelziside B, 14-deoxyandrographolide	In vivo, in vitro	ERK 1/2, JNK, p38 MAPK inhibition, prevent apoptosis	[58]
*Matricaria chamomilla* (Chamomille)	*α*-Bisabolol	In vitro, in vivo	MAPK pathway regulation	[26]
*Amaryllidaceae*, *Asparagaceae*, *Asteraceae*, *Dioscoreaceae*, *Fabaceae*, *Liliaceae*, *Plantaginaceae*, *Smilacaceae*, *Solanaceae*, *Zygophyllaceae* families	Triterpenoid glycosides derived saponins	In vivo	↓ Cytoplasmic toxic compounds	[65]
*Astragalus* spp., *Ginkgo biloba* (Ginkgo), *Panax pseudoginseng* (Notoginseng), *Phyllanthus emblica* (Emblic)	Astragaloside IV, diosgenin, ginsenoside Re, lupeol, oleanolic acid, phylloemblycin B, 20(S)-protopanaxtriol, ursolic acid	In vivo	↓ MMP13 and MMP14 expression	[66]
*Artemisia annua* (Sweet wormwood), *Betula* spp., *Gardenia jasminoides* (Gardenia), *Hypopterygium* gns., *Lonicera japonica* (Japanese honeysuckle), *Panax ginseng* (Asian ginseng), *Rabdosia rubescens* (Donglingcao), *Tripterygium wilfordii* (Thunder duke vine)	Artemisinin, betulin, celastrol, dioscina, geniposide, ginsenoside Rg3, oridonin, sweroside, triptolide	In vivo	Caspases-1 inhibition	[24]
*Aralia* spp.	Elatoside F, araloside C, chikusetsusaponin IVa, chikusetsusaponin IVa, elatoside I, oleanolic acid 3-O-*β*-D-glucopyranosyl(1 → 3)-*α*-L-rhamnopyranosyl (1 → 2)-*α*-L-arabinopyranoside, kaurenoic acid, continental acid, 7-oxo-ent-pimara-8 (14), 15-dien-19-oic acid, 3-O-{*β*-D-glucopyranosyl-(1 → 2)-[*β*-D-glucopyranosyl -(1 → 3)]-*β*-D-glucuronpyranosyl} 28-O-*β*-D-glucopyranosyl ester of oleanolic acid, 3-O-{*β*-D-glucopyranosyl-(1 → 2)-[*β*-D-glucopyranosyl-(1 → 3)]-*β*-D-glucuronpyranosyl}olean-11,13(18)-diene-28-oic ester 28-O-β-D-glucopyranosyl	In vivo	Apoptosis prevention due to antioxidant properties	[67]
*Salvia* spp.	Ferruginol	In vitro	Cardiotoxicity protection	[88]
*Artemisia annua* (Sweet wormwood), *Centella asiatica* (Indian pennywort), *Ginkgo biloba* (Ginkgo), *Lamiaceae* family, *Ligustrum lucidum* (Chinese privet), *Olea europaea* (Olive), *Swertia mussotii* (Zangyinchen)	Asiatic acid, oleanolic acid, ursolic acid, artemisinin, ginkgolide B	In vitro, in vivo	MAPK inhibition, stress apoptosis inhibition through the PI3K/AKT/mTOR path	[84]
*Ziziphora clinopodioides* subsp. *bungeana*	Ziziphoric acid, ziziphoroside D, 6′-malonylzizi-phoroside A	In vivo	Coronary artery problems protection	[89]
*Polygonatum sibiricum* (Siberian Solomon’s Seal)	Curcumenol, geniposide	In vitro, in vivo	↓ Bax and Cleaved Caspase-3 ↑ Bcl-2	[85]
*Lagerstroemia speciosa* (*Banaba*)	Corosolic acid, ursolic acid	In vivo	↓ Bax and Cleaved Caspase-3 ↑ Bcl-2	[47]
*Cleome viscosa*	Lupeol acetate	In silico	↓ Bax and Cleaved Caspase-3 ↑ Bcl-2 (via PI3K-Akt activation)	[90]

↓ reduce; ↑ activate.

### 2.2. Anti-Remodeling Effect

This section examines the anti-remodeling effect of compounds after ischemia. A total of ten articles were found (Table 4).

Cardiac remodeling refers to structural changes in the heart in response to various conditions and plays a critical role in cardiovascular diseases. While it initially serves to compensate for the loss of cardiac function caused by heart damage, these compensatory mechanisms can become detrimental, potentially leading to life-threatening conditions such as heart failure and fatal arrhythmia. Cardiac remodeling can be classified into two types: (1) Physiological remodeling, which responds to the physiological growth of the heart to meet the demand requirements of several physiological conditions such as pregnancy or exercise. (2) Pathological remodeling or pathological hypertrophy in response to several factors such as hypertension, myocardial injury or neurohumoral activation, often contributing to disease progression [91].

In myocardial ischemia, coronary artery blood flow is cut off, depriving cardiomyocytes of nutrients and oxygen [92]. Over time, oxidative stress occurs in cardiomyocytes due to nutrient and oxygen deprivation, which triggers the activation of apoptotic pathways and the gradual death of ischemic tissue [92,93]. During reperfusion, the tissue also suffers from various difficulties, such as necrosis, oxidative stress, inflammation, alteration of calcium homeostasis and endothelial dysfunction [94,95]. After injury, cardiac fibroblasts are activated and differentiated into myofibroblasts (myoFbs), the key mediators in pathological remodeling. MyoFbs develop proliferative and secretory activities contributing to collagen deposition, responsible for creating fibrotic scars and eventually cardiac dysfunction [96]. This scar tissue, being less resistant and elastic, begins to deform due to the contraction of the heart and the increased pressure of blood inside the chambers with each beat. Over time, the shape of the heart becomes further deformed by the scarred area, impairing its function and even causing the scar tissue to rupture, leading to death [92,93].

Some compounds provide protection by preparing the cells to reduce damage when ischemia occurs until blood flow to the area is restored [16]. Other compounds can improve tissue architecture to make the necrotic tissue more resistant when ischemia occurs [78]. There are also compounds with the ability to inhibit organ hypertrophy and fibrosis, reducing the negative effects of cardiac remodeling [79].

Once different articles have been reviewed, *Artemisia annua*, *Betula*, *Gardenia jasminoides*, *Hypterygium*, *Lonicera japonica*, *Panax ginseng*, *Rabdosia rubescens* and *Tripterygium wilfordii* should be mentioned since the combined effect of all of them makes preventing and reducing ischemia achievable, in addition to fibrosis and hypertrophy of the heart to maintain its shape [24]. Also worth mentioning is the plant *Andrographis paniculata*, since it has been observed that its compounds interact with the ERK1/2 pathways, allowing the scar tissue to have a better structure and less likelihood of distension [78].

Triterpenes are also good candidates to treat heart failure due to their anti-remodeling effect. Two examples are ursolic and oleanoic acid, with proven antifibrotic activity. It is well known that collagen deposition is induced by TNF-*α* and TGF-*β* pathways, and the decrease in enzymes such as MMP-2 (Matrix MetalloProteinase) involved in collagen matrix degradation contributes to cardiac fibrosis progression. The triterpenes mentioned above have proven antifibrotic activity by decreasing TNF-*α* and TGF-*β* levels and increasing MMP-2 expression [32,33,49,97]. It could explain the anti-remodeling effect of *Artemisia annua*, *Centella asiática*, *Ginkgo biloba*, *Lamiaceae* family, *Ligustrum lucidum*, *Olea europaea* and *Swertia mussotii*, plants with a composition rich in both triterpenes. Another triterpene, betulin, also mitigates cardiac hypertrophy and fibrosis [48].

**Table 4:** Studies demonstrating the anti-remodeling effect.

Plant	Terpenoids	Study	Effect	Reference
*Origanum vulgare* (Oregano), *Thymus vulgaris* (Common thyme)	Carvacrol	In vitro, in vivo	↓ Tissue necrosis in pretreatment	[16]
*Andrographis paniculata* (Creat)	Andrographatoside, oleanolic acid, andrographolide, 3-O-*β*-D-glucopyranosilandrographolide, 3-oxo-14-deoxy-11,12-didehydroandrographolide, neoandrographolide, 14-deoxyandrographolide, andrograpanin, 3-O-*β*-D-glucosyl-14 -deoxyandrographolide, 6′-acetylneoandrographolide, 14-deoxy-17-*β*-hydroxyandrographolide, 19-O-[*β*-D-apiofuranosyl(1 → 2)-*β*-D-glucopyranoyl]-3,14 dideoxyandrographolide, isoandrographolide, 14-Deoxy-11-oxo-andrographolide, 14-Deoxy-12-hydroxyandrographolide, 8,17-epoxy-14-deoxyandrographolide, 3-O-*β*-D-glucosyl-14-deoxyandrographolide, 12S-hydroxyandrographolide, 14-Deoxy-15-isopropylidene-11,12-didehydroandrographolide, bisandrographolide A, bisandrographolide B, bisandrographolide C, bisandrographolide D, bisandrographolide E, bisandrographolide F, bisandrographolide G and bisandrographolide ether	In vivo	ERK1/2 path regulation to improve tissue structure	[78]
*Isodon rubescens* (Donglingcao)	Oridonin	In vitro, in vivo	Reperfusion effectts mitigation	[79]
*Ceriops decandra* (Clumped yellow mangrove)	Isosteviol	In vivo	Heart morphology maintained	[17]
*Scrophularia ningpoensis* (Ningpo figwort)	Sugiol, lupeol, ursolic acid, oleanonic acid, eucalyptolic acid, scrokoelziside A, scrokoelziside B, 14-deoxy andrographolide	In vivo	ERK 1/2 and p38 MAPK pathway inhibition	[58]
*Matricaria chamomilla* (Chamomille)	*α*-Bisabolol	In vitro, in vivo	↓ Ischemia effects	[26]
*Ginkgo biloba* (Ginkgo)	Ginkgolides	In vitro, in vivo	Col I, Col III and fibronectine regulation that ↓ tissue fibrosis	[98]
*Amaryllidaceae*, *Asparagaceae*, *Asteraceae*, *Dioscoreaceae*, *Fabaceae*, *Liliaceae*, *Plantaginaceae*, *Smilacaceae*, *Solanaceae*, *Zygophyllaceae* families	Triterpenoid glycosides derived saponins	In vivo	Angiotensin I inhibition to ↓ hypertrophy	[65]
*Artemisia annua* (Sweet wormwood), *Betula* spp., *Gardenia jasminoides* (Gardenia), *Hypopterygium* gns., *Lonicera japonica* (Japanese honeysuckle), *Panax ginseng* (Asian ginseng), *Rabdosia rubescens* (Donglingcao), *Tripterygium wilfordii* (Thunder duke vine)	Artemisinin, betulin, celastrol, dioscina, geniposide, ginsenoside Rg3, oridonin, sweroside, triptolide	In vivo	Ischemia prevention + ↓ ischemia effects + angiotensin II inhibition to reduce fibrosis + ↓ hypertrophy	[24]
*Falcaria vulgaris* (Sickleweed)	*α*-Pinene, spathulenol, carvacrol, limonene	In vitro, in vivo	↑ and maintain coronary artery Flow	[70]
*Artemisia annua* (Sweet wormwood), *Centella asiatica* (Indian pennywort), *Ginkgo biloba* (Ginkgo), *Lamiaceae* family, *Ligustrum lucidum* (Chinese privet), *Olea europaea* (Olive), *Swertia mussotii* (Zangyinchen)	Asiatic acid, oleanolic acid, ursolic acid, artemisinin, ginkgolide B	In vitro, in vivo	↓ Myocardial hypertrophy	[84]
*Polygonatum sibiricum* (Siberian Solomon’s Seal)	Curcumenol, geniposide	In vitro, in vivo	↓ Myocardial hypertrophy through AMPK*α* activation and mTOR, ERK inhibition	[85]

↓ reduce; ↑ activate.

### 2.3. Antiarrhythmia Effect

This section discusses the antiarrhythmic effect and its properties for maintaining heart rhythm. A total of ten articles were found (Table 5).

The heart is an autonomous organ, due to its intrinsic rhythmic activity, regulated by the autonomic nervous system. It has its own pacemaker that regulates heart rate and proper myocardial contraction [99]. The impulse is generated in the sinoatrial node, which rhythmically generates stimuli and propagates to the atrioventricular (AV) node where it is delayed, allowing for maximum contraction of the atria. Finally, the impulse retained in the AV node travels to the ventricles through the bundle of His and the Purkinje fibers, generating ventricular contraction. Arrhythmias occur when some of the pacemaker components fail to perform their function properly, generating irregular impulses and arrhythmic contractions [99].

Arrhythmic manifestations include tachycardia, bradycardia and other rhythm disorders. Uncontrolled arrhythmia can lead to syncope and sudden death. ECG alterations such as long and short QT syndrome are due to dysfunction of ion channel activity (congenital mutations or drug-induced) and predispose individuals to developing malignant arrhythmia [100]. It has been shown that some botanical compounds are capable of rectifying arrhythmia by regulating and blocking calcium channels, which are the channels responsible for the action potential plateau [15,101]. The monoterpene (-)-carvone showed an antiarrhythmic role in whole hearts, reducing the severity of ventricular fibrillation. This effect could be explained by the blockade of L-type Ca^2+^ channel (LTCC) and Ca^2+^ transient amplitude, both promoting a negative inotropic effect on atrial and ventricular contraction [101]. LTCC blockers belong to class IV antiarrhythmic drugs and are widely used in clinical practice, with verapamil being the gold-standard of class-IV antiarrhythmic drugs. (-)-carvone has a similar effect to verapamil with the advantage of being partially irreversible [99]. A similar effect was observed with the ketone monoterpene R (+)-pulegone, found in plants species of *Mentha* genus [102]. Besides the negative inotropic effect of R(+)-pulegone, class III-antiarrhythmic behavior has been shown for this compound, able to interfere with the repolarizing potassium channels such as I_Kr_ (rapid delayed rectifier potassium current), I_to_ (transient outward potassium current), I_K1_ (inward rectifier potassium current) and I_ss_ (steady state potassium current), blocking its activity in a fast and reversible way. This results in an increase in action potential duration (APD) and therefore ECG QT interval [103].

Finally, a plant extract with promising antiarrhythmic properties is storax. It has been shown to block the Kir2.1 potassium rectifier channel (IK1). The storax component underlying this action could be hydrocinnamic acid, and it could be a therapeutic approach to treat type 3 short QT syndrome, a rare genetic condition caused by “gain-of-function” mutations of *kir2.1* [55,104].

The triterpene celastrol also demonstrated antiarrhythmic properties. It reduces susceptibility to ventricular arrhythmias by alleviating fibrosis through inflammasome suppression (inhibition of the NLRP3/Caspase-1 pathway) and by improving ventricular electrical properties via upregulation of Cx43 expression. The pro arrhythmogenic role of pyroptosis is explained by the downregulation of Cx43 induced by p38 phosphorylation, which is triggered by inflammatory molecules such as IL 1β, released during pyroptosis. By preventing pyroptosis, celastrol avoids IL-1*β* mediated p38 activation and consequently prevents Cx43 downregulation [105].

Having reviewed all the properties of the selected species, it is worth highlighting that *Eleutherococcus* species are particularly interesting in the context of arrhythmia. *Eleutherococcus* can protect against substances that cause arrhythmia, such as BaCl_2_, CaCl*_2_*, aconite or the Langendorff method [64].

**Table 5:** Studies demonstrating the antiarrhythmic effect.

Plant	Terpenoids	Study	Effect	Reference
*Alpinia zerumbet* (Shell ginger), *Cymbopogon citratus* (Lemon grass), *Cymbopogon winterianus* (Java citronella), *Eucalyptus* spp., *Lippia alba* (Bushy matgrass), *Mentha villosa* (Hairy mint), *Origanum vulgare* (Oregano), *Thymus vulgaris* (Common thyme)	Carvacrol, citronellol, eucalyptol, (-)-linalool, (+)-linalool, menthol, myrtenal, myrtenol, rotundifolone, sobrerol, thymol, *α*-limonene, *α*-terpinen-4-ol, *α*-terpineol, *p*-cymene, peryl alcohol, *α*-pinene, *β*-pinene	In vitro, in vivo	Ca^2+^ channels regulation	[15]
*Mentha pulegium* (Pennyroyal)	R(+)-pulegone	In vivo	K^+^ flow regulation	[103]
*Liquidambar orientalis* (Oriental sweetgum)	Oleanolic acid, betulinic acid, corosolic acid, maslinic acid, epibetulinic acid, betulonic acid	In vitro, in vivo	Mutated Kir2.1 rectifier K^+^ channel blocked	[55]
*Melissa officinalis* (Lemon balm)	Citronellal, thymol, citral, *β*-caryophyllene, caryophyllene oxide, limonene, germacrene, ursolic acid, oleanolic acid	In vivo	Ca^2+^ channels regulation + QRS, QTc, JT and TpTe intervals regulation	[59]
*Eleutherococcus* spp.	Eleutherococcus lupane triterpenes	In vitro, in vivo	Protection against arrhythmias caused by Langendorff method, BaCl_2_, CaCl_2_ and toxoside	[64]
*Artemisia annua* (Sweet wormwood), *Betula* spp., *Gardenia jasminoides* (Gardenia), *Hypopterygium* gns., *Lonicera japonica* (Japanese honeysuckle), *Panax ginseng* (Asian ginseng), *Rabdosia rubescens* (Donglingcao), *Tripterygium wilfordii* (Thunder duke vine)	Artemisinin, betulin, celastrol, dioscina, geniposide, ginsenoside Rg3, oridonin, sweroside, triptolide	In vivo	Aconitine’s arrhythmias protection	[24]
*Aralia* spp.	Elatoside F, araloside C, chikusetsusaponin IVa, chikusetsusaponin IVa, elatoside I, oleanolic acid 3-O-*β*-D-glucopyranosyl(1 → 3)-*α*-L-rhamnopyranosyl (1 → 2)-*α*-L-arabinopyranoside, kaurenoic acid, continental acid, 7-oxo-ent-pimara-8 (14), 15-dien-19-oic acid, 3-O-{*β*-D-glucopyranosyl-(1 → 2)-[*β*-D-glucopyranosyl-(1 → 3)]-*β*-D-glucuronpyranosyl} 28-O-*β*-D-glucopyranosyl ester of oleanolic acid, 3-O-{*β*-D-glucopyranosyl-(1 → 2)-[*β*-D-glucopyranosyl-(1 → 3)]-*β*-D-glucuronpyranosyl}olean-11,13(18)-diene-28-oic ester 28-O-*β*-D-glucopyranosyl	In vivo	↓ MMP-9 expression + relaxing arterial smooth muscle	[67]
*Cucurbitaceae* family	Cucurbitacin triterpenoids	In vitro, in vivo	Doxorubicin’s arrhythmias protection	[69]
*Panax notoginseng* (Chinese ginseng)	*Panax notoginseng* saponins	In vivo	K^+^ and Ca^2+^ channels regulation	[83]
*Bauhinia* spp.	23-hydroxy-3*α*-[O-*α*-L-1C4-rhamnopyranosyl-(1″→4′)-O-*α*-L-4C1-arabinopyranosyl-oxy]olean-12-en-28-oic acid O-*α*-L-1C4-rhamnopyranosyl-(1⁗′ → 4⁗)-O-*β*-D-4C1-glucopyranosyl-(1⁗→6‴)-O-*β*-D-4C1-glucopyranosyl ester, champine A	In vivo, in vitro	Arrhythmia control in animal model	[86]

↓ reduce.

### 2.4. Antihypertensive Effect

This section addresses antihypertensive and arterial-relaxing effects. A total of fourteen articles were found (Table 6).

Hypertension is the increase in pressure within the arteries exerted by the blood [106]. Increased blood pressure can occur for two reasons: increased blood flow or narrowing of the artery lumen. Narrowing of the artery lumen is mainly caused by increased vascular contraction and arterial remodeling, both processes regulated by complex cell signaling pathways involving nervous and immune systems [107]. High blood pressure is a silent heart problem, as the disease can be present without any symptoms. Due to increased resistance in the arteries, the heart must contract with greater effort, which can cause cardiomyocyte hypertrophy and the corresponding problems [106].

Aside from the problems caused by hypertension, as discussed in previous sections, high blood pressure following ischemia and scarring of part of the heart tissue are detrimental to the proper functioning of the myocardium. This increase results in greater pressure and force exerted by the blood on the scar tissue, causing greater distension and the likelihood of rupture [93].

The properties of pressure-reducing compounds are based on their ability to directly interfere with the molecules that activate smooth muscle contraction, such as angiotensin II, as well as with ion channels involved in artery relaxation or NO-cGMP pathway [15,57]. Terpenes such as carvacrol, thymol and rotundifolone can induce endothelium-independent relaxation, mainly through inhibition of extracellular Ca^2+^-influx or Ca^2+^ release from the sarcoplasmic reticulum (SR). The resulting decrease in intracellular Ca^2+^ level reduces the sensitivity of the contractile machinery to Ca^2+^, leading to vascular relaxation [108].

Other compounds, such as the monoterpenes eucalyptol and α-terpineol and the triterpene betulinic acid, exert their vasorelaxant action through an endothelium-dependent pathway that requires activation of NO-cGMP signaling pathway [109] and are mediated through eNOS activation and increased NO levels [44]. In addition, carvacrol can induce another form of endothelium-mediated relaxation by increasing intracellular Ca^2+^ levels in endothelial cells, which leads to activation of Ca^2+^-sensitive K^+^ channels (I_KCa_ and S_KCa_). Activation of these channels causes hyperpolarization of the vascular smooth muscle cells membrane, resulting in vasodilation [110].

Besides the terpenoids previously mentioned, several studies have reported antihypertensive effects of some plant extracts such as *Matricaria chamomilla* and *Liquidambar orientalis*, both of which exhibit promising properties. Alcohol, phenolic and oil extracts of *Matricaria chamomilla* have shown antihypertensive effect in both normotensive and hypertensive rats, with the phenolic extract being the more promising. This effect could be attributed to its inhibitory action on Angiotensin Converting Enzyme (ACE) [111]. The sesquiterpene *α*-bisabolol, originally isolated from this plant, may be responsible for this activity [26].

**Table 6:** Studies demonstrating the antihypertensive effect.

Plant	Terpenoids	Study	Effect	Reference
*Alpinia zerumbet* (Shell ginger), *Cymbopogon citratus* (Lemon grass), *Cymbopogon winterianus* (Java citronella), *Eucalyptus* spp., *Lippia alba* (Bushy matgrass), *Mentha villosa* (Hairy mint), *Origanum vulgare* (Oregano), *Thymus vulgaris* (Common thyme)	Carvacrol, citronellol, eucalyptol, (-)-linalool, (+)-linalool, menthol, myrtenal, myrtenol, rotundifolone, sobrerol, thymol, *α*-limonene, *α*-terpinen-4-ol, *α*-terpineol, *p*-cymene, peryl alcohol, *α*-pinene, *β*-pinene	In vitro, in vivo	Ca^2+^ channels interaction + TRPM8 activation + ↓ NO via NO-cGMP	[15]
*Juniperus phoenicea* (Sabina negral), *Laurus nobilis* (Laurel), *Melaleuca armillaris* (Bracelet honey myrtle), *Thymbra capitata* (Andalusian thyme)	*α*-Pinene, *β*-myrcene, *p*-cymene, *γ*-terpinene, *β*-caryophyllene, *β*-elemene, *α*-himachalene, *γ*-muurolene, *β*-caryophyllene oxide, thymol	In vitro, in vivo	Connection between vasorelaxation and antioxidants	[52]
*Matricaria chamomilla* (Chamomille)	Sterols and triterpenes of *Matricaria chamomilla*	In vitro, in vivo	Inhibitory action on ACE	[111]
*Liquidambar orientalis* (Oriental sweetgum)	Oleanolic acid, betulinic acid, corosolic acid, maslinic acid, epibetulinic acid, betulonic acid	In vitro, in vivo	Coronary artery dilation	[55]
*Trypterygiun wilfordii* (Thunder duke vine), *Salvia milthiorrhiza* (Red sage)	Celastrol, cryptotanshinone, geraniol, lycopene, oleanolic acid, thymoquinone, ursolic acid	In vitro, in vivo	↓ NO and endothelin 1 + ↑ PGI_2_ increase	[77]
*Nigella sativa* (Black cumin)	Thymoquinone, carvacrol, 4-terpineol, *α*-pinene, thymol, t-anethole, thymohydroquinone, ithymoquinone, *p*-cymene	In vitro, in vivo	↓ Angiotensin II	[57]
*Scrophularia ningpoensis* (Ningpo figwort)	Sugiol, lupeol, ursolic acid, oleanonic acid, eucalyptolic acid, scrokoelziside A, scrokoelziside B, 14-deoxy andrographolide	In vivo	↓ Angiotensin II, thromboxane B2, endothelin 1	[58]
*Melissa officinalis* (Lemon balm)	Citronellal, thymol, citral, β-caryophyllene, caryophyllene oxide, limonene, germacrene, ursolic acid, oleanolic acid	In vivo	Ca^2+^ channels interaction	[59]
*Matricaria chamomilla* (Chamomille)	*α*-Bisabolol	In vitro, in vivo	↓ α-glucosidase, angiotensin converting enzymes, beta-2 adrenergic receptors, glucocorticoids, HMG-CoA reductase, insulin, mineralocorticoids, potassium channels and peroxisome proliferator-activated receptor alpha interaction + ↓ smooth muscle contraction	[26]
*Eleutherococcus* spp.	Eleutherococcus lupane triterpenes	In vitro, in vivo	Angiotensin converting enzyme inhibition	[64]
*Amaryllidaceae*, *Asparagaceae*, *Asteraceae*, *Dioscoreaceae*, *Fabaceae*, *Liliaceae*, *Plantaginaceae*, *Smilacaceae*, *Solanaceae*, *Zygophyllaceae* families	Triterpenoid glycosides derived saponins	In vivo	Potassium channels regulation	[65]
*Aralia* spp.	Elatoside F, araloside C, chikusetsusaponin IVa, chikusetsusaponin IVa, elatoside I, oleanolic acid 3-O-*β*-D-glucopyranosyl(1 →3)-*α*-L-rhamnopyranosyl (1 → 2)-*α*-L-arabinopyranoside, kaurenoic acid, continental acid, 7-oxo-ent-pimara-8 (14), 15-dien-19-oic acid, 3-O-{*β*-D-glucopyranosyl-(1 → 2)-[*β*-D-glucopyranosyl-(1 → 3)]-*β*-D-glucuronpyranosyl} 28-O-*β*-D-glucopyranosyl ester of oleanolic acid, 3-O-{*β*-D-glucopyranosyl-(1 → 2)-[*β*-D-glucopyranosyl-(1 → 3)]-*β*-D-glucuronpyranosyl}olean-11,13(18)-diene-28- oic ester 28-O-*β*-D-glucopyranosyl	In vivo	Vasorelaxant effect	[67]
*Falcaria vulgaris* (Sickleweed)	*α*-Pinene, spathulenol, carvacrol, limonene	In vitro, in vivo	Increase coronary fluid flow	[70]
*Panax notoginseng* (Chinese ginseng)	*Panax notoginseng* saponins	In vivo	Ca^2+^ channels regulation	[83]

↓ reduce; ↑ activate.

### 2.5. Anti-Atherosclerosis Effect

This section addresses the anti-atherosclerotic effects including the reduction of arterial blockages and the improvement in blood flow. A total of ten articles were found (Table 7).

Atherosclerosis is a condition that thickens and hardens the walls of the arteries, causing them to lose elasticity [112]. It is a multifactorial disease responsible for many CVDs caused by the deposition of lipids between the layers of the arteries, with hypercholesterolemia, or elevated blood cholesterol, being the primary contributing factor. Accumulation of cholesterol within the arterial wall narrows it, increasing blood pressure and the risk of obstruction. The loss of arterial elasticity also makes the vessel more prone to rupture under elevated pressure. Among all affected arteries, coronary arteries are of particular importance because their small diameter and thin walls make them especially susceptible to blockage or rupture, potentially resulting in cardiovascular ischemia [112].

Some compounds have been observed to act as preventive agents by decreasing the levels and oxidation of low-density lipoprotein (LDL) and very low-density lipoprotein (VLDL), in addition to increasing high-density lipoprotein (HDL). This reduces or stops the buildup of cholesterol in the arteries, preventing atherosclerosis [55,113]. Meanwhile, other compounds reduce inflammation caused by atherosclerosis, preventing the worsening of circulation [67,88]. There are compounds with the ability to increase the cholesterol efflux in the blood, preventing its accumulation in arterial walls [66]. Additionally, some compounds have been found to prevent abnormal development of smooth muscles of the arteries by suppressing MMP-9 [67].

After reviewing the information reported in various studies, *Liquidambar orientalis* and *Urtica dioica* exhibit particularly beneficial effects in this context. On the one hand, storax from *Liquidambar orientalis* prevents LDL oxidation; this property is very remarkable given the central role of oxidized LDL in the formation and accumulation of foam cells within atherosclerotic plaques. Its anti-atherosclerotic effect is further supported by its ability to reduce blood viscosity, thereby improving blood flow [55]. The triterpene maslinic acid could be responsible for anti-atherosclerotic properties attributed to *Liquidambar orientalis*, since experimental results have shown its ability to prevent foam cell formation [114,115] and significant antihyperlipidemic potential [116]. The antihyperlipidemic role of maslinic acid is a common property of triterpenes. A similar effect was shown for lupeol and lupeol linolate in Wistar rats fed a high-cholesterol diet [117].

Conversely, *Urtica dioica* improves the balance of LDL and HDL, increasing HDL levels and lowering LDL levels, in addition to enhancing cholesterol utilization and maintaining lipids within normal values [71].

**Table 7:** Studies demonstrating the anti-atherosclerotic effect.

Plant	Terpenoids	Study	Effect	Reference
*Liquidambar orientalis* (Oriental sweetgum)	Oleanolic acid, betulinic acid, corosolic acid, maslinic acid, epibetulinic acid, betulonic acid	In vitro, in vivo	↓ LDL oxidation + ↓ blood viscosity	[55]
*Senna auriculata* (Avaram senna)	Oleanolic acid	In vitro, in vivo	↓ LDL oxidation	[56]
*Nigella sativa* (Black cumin)	Thymoquinone, carvacrol, 4-terpineol, *α*-pinene, thymol, t-anethole, thymohydroquinone, ithymoquinone, *p*-cymene	In vitro, in vivo	↓ LDL and TGs + ↑ HDL increase	[57]
*Eleutherococcus* spp.	Eleutherococcus lupane triterpenes	In vitro, in vivo	↓ Coronary angina symptoms	[64]
*Amaryllidaceae*, *Asparagaceae*, *Asteraceae*, *Dioscoreaceae*, *Fabaceae*, *Liliaceae*, *Plantaginaceae*, *Smilacaceae*, *Solanaceae*, *Zygophyllaceae* families	Triterpenoid glycosides derived saponins	In vivo	↑ Cholesterol flow	[65]
*Artemisia annua* (Sweet wormwood), *Betula* spp., *Gardenia jasminoides* (Gardenia), *Hypopterygium* gns., *Lonicera japonica* (Japanese honeysuckle), *Panax ginseng* (Asian ginseng), *Rabdosia rubescens* (Donglingcao), *Tripterygium wilfordii* (Thunder duke vine)	Artemisinin, betulin, celastrol, dioscina, geniposide, ginsenoside Rg3, oridonin, sweroside, triptolide	In vivo	↓ LDL + ↑ HDL	[24]
*Aralia* spp.	Elatoside F, araloside C, chikusetsusaponin IVa, chikusetsusaponin IVa, elatoside I, oleanolic acid 3-O-*β*-D-glucopyranosyl(1 → 3)-*α*-L-rhamnopyranosyl (1 → 2)-*α*-L-arabinopyranoside, kaurenoic acid, continental acid, 7-oxo-ent-pimara-8 (14), 15-dien-19-oic acid, 3-O-{*β*-D-glucopyranosyl-(1 → 2)-[*β*-D-glucopyranosyl-(1 → 3)]-*β*-D-glucuronpyranosyl} 28-O-*β*-D-glucopyranosyl ester of oleanolic acid, 3-O-{*β*-D-glucopyranosyl-(1 → 2)-[*β*-D-glucopyranosyl-(1 → 3)]-*β*-D-glucuronpyranosyl}olean-11,13(18)-diene-28-oic ester 28-O-*β*-D-glucopyranosyl	In vivo	Smooth muscle overgrowth prevented	[67]
*Salvia* spp.	Ferruginol	In vitro	Lipids disposition in plaque decrease	[88]
*Urtica dioica* (Nettle)	4,7-Megastigma-diene-3, 9-diol; (3S,6R,7E,9R)-form, 3-ketone, 9-O-[ *β* -D-glucopyranosyl-(1⟶2)-*β*-D-glucopyranoside], 1-(3, 4-dihydroxyphenyl)-1, 2-propanediol; 3′-Me ether, (9Z,11E)-1, 3-hydroxy-9, 11-octadeca-dienoic acid, hexahydrofarnesyl acetone, geranyl acetone, (E)-anethole, *p*-hydroxybenzaldehyde, b-ionone	In vitro, in vivo	Blood lipid levels improve + ↓ cholesterol levels + ↓ LDLs/HDLs	[71]
*Petroselinum crispum* (Parsley)	*m*-Cymenene, *p*-cymenene, 1,3,8-*p*-menthatriene, trans-borneol, camphene, carvacrol, cryptone, fenchyl alcohol, *β*-damascenone, limonene, linalool, linalool acetate, mentol, myrcene, myrtenal, p-cymene, pulegone, sabinene, terpinolene, tricyclene, *α*-phellandrene, *α*-pinene, *α*-terpinene, *α*-terpineol, *α*-thujene, (Z)- and (E)-*β*-ocimene, *β*-phellandrene, *β*-pinene, *γ*-terpinene, menthone, *β*-terpinyl acetate, trans-*α*-bergamotene, *β*-bourbonene, carotol, *α*-amorphene, *β*-duprezianene, cis-thujopsene, copaene, copaen-15-ol, *β*-copaen-4*α*-ol, germacrene B, germacrene D, *α*-muurolene, *γ*-muurolene, isoaromadendrene epoxide, nerolidol, ledene oxide-(II), selin-11-en-4*α*-ol, *β*-bisabolene, trans-*β*-farnesene, (+)-epi-bicyclosesquiphellandrene, *α*-selinene, *β*-selinene, *β*-caryophyllene, caryophylla-4(12),8(13)-dien-5β-ol, caryophyllene oxide, *β*-elemene, *γ*-elemene, *α*-cadinol, t-cadinol, *α*-cadinene, *γ*-cadinene, *α*-cedrene	In vivo	↓ ALT, ALP, AST enzymes	[118]

↓ reduce; ↑ activate.

### 2.6. Antidiabetic Effect

This section focuses on understanding the antidiabetic effects and their correlation with heart problems. A total of twelve articles were found (Table 8).

Diabetes is known to exacerbate cardiovascular diseases, with the coronary arteries being particularly affected [119]. Elevated blood glucose and lipid levels in diabetes contribute to the formation of atherosclerotic plaques within the arteries. This increases the risk of ischemia and associated cardiovascular complications. Effective management of diabetes and maintenance of normal blood glucose levels can help to mitigate the harmful effects of the disease on the heart [119].

Some compounds, such as the pentacyclic triterpenoid oleanolic acid, present in the aerial parts of *Senna auriculata*, exert hypoglycemic effects in STZ-induced diabetic rats [120]. This compound can increase insulin levels by stimulating secretion from pancreatic *β*-cells and preventing *β*-cells destruction [121], thereby protecting against spikes in blood glucose that could otherwise contribute to arterial plaque formation [56]. Other compounds, such as triterpene saponins, reduce intestinal sugar absorption by inhibiting the enzymes *α*-amylase and *α*-glucosidase, thereby decreasing carbohydrate digestion and lowering blood sugar levels [56]. Compounds that protect β-cells from apoptosis have also been identified. One example is the monoterpene thymoquinone, whose anti-apoptotic action is characterized by increased antioxidant enzyme expression and inhibition of inflammatory response. In addition, by regulating the expression of survival-related genes, thymoquinone inhibits the apoptotic stress response induced by *β*-cell overstimulation under high blood glucose conditions [122]. Furthermore, certain compounds have been shown to improve insulin response and resensitize insulin receptors. They have been observed to do so by interacting with and modulating AMPK activity [64].

Among all the species with antidiabetic properties, *Senna auriculata* and *Falcaria vulgaris* stand out together with *Acanthopanax senticosus*. The first two species exhibit very similar effects: they reduce glucose absorption, lower blood glucose levels, protect the pancreatic β-cells responsible for insulin production, and stimulate insulin release, thereby accelerating the normalization of blood glucose levels [56,70]. The root of the third plant (*Acanthopanax senticosus*) has long been used in traditional Chinese medicine for the treatment of diabetes. In addition, there is scientific evidence supporting the antidiabetic properties of its fruit (Goka fruit), which improves insulin resistance and hepatic lipid accumulation in obese mice by activating the enzyme AMPK, involved in the regulation of liver gluconeogenesis and lipogenesis [123].

Diabetic cardiomyopathy is alleviated by the triterpenes ursolic and oleanoic acids due to their anti-remodeling properties described previously [43,124].

**Table 8:** Studies demonstrating the antidiabetic effect.

Plant	Terpenoids	Study	Effect	Reference
*Senna auriculata* (Avaram senna)	Oleanolic acid	In vitro, in vivo	↑ Insulin levels + ↓ glucose absorption + *β*-cells protection	[56]
*Andrographis paniculata* (Creat)	Andrographatoside, oleanolic acid, andrographolide, 3-O-*β*-D-glucopyranosilandrographolide, 3-Oxo-14-deoxy-11,12-didehydroandrographolide, neoandrographolide, 14-deoxyandrographolide, andrograpanin, 3-O-*β*-D-glucosyl-14 -deoxyandrographolide, 6′-acetylneoandrographolide, 14-deoxy-17-*β*-hydroxyandrographolide, 19-O-[*β*-D-apiofuranosyl(1 → 2)-*β*-D-glucopyranoyl]-3,14 dideoxyandrographolide, isoandrographolide, 14- deoxy-11-oxo-andrographolide, 14-deoxy-12-hydroxyandrographolide, 8,17-epoxy-14-deoxyandrographolide, 3-O-*β*-D-glucosyl-14-deoxyandrographolide, 12S-hydroxyandrographolide, 14-deoxy-15-isopropylidene-11,12-didehydroandrographolide, bisandrographolide A, bisandrographolide B, bisandrographolide C, bisandrographolide D, bisandrographolide E, bisandrographolide F, bisandrographolide G, bisandrographolide ether	In vivo	↓ Blood glucose levels	[78]
*Nigella sativa* (Black cumin)	Thymoquinone, carvacrol, 4-terpineol, *α*-pinene, thymol, t-anethole, thymohydroquinone, ithymoquinone, *p*-cymene	In vitro, in vivo	*β* -cells protection	[57]
*Scrophularia ningpoensis* (Ningpo figwort)	Sugiol, lupeol, ursolic acid, oleanonic acid, eucalyptolic acid, scrokoelziside A, scrokoelziside B, 14-deoxy-andrographolide	In vivo	↑ Insulin levels + ↓ blood glucose	[58]
*Bougainvillea glabra* (Lesser bouginvillea)	Oleananoic acid acetate, oleoside dimethyl ester, goyaglucoside, oleanolic acid 3-O-beta-D-glucosiduronic acid, phytol, squalene, geranylgeraniol	In vitro	↓ Blood glucose levels	[60]
*Eleutherococcus* spp.	Eleutherococcus lupane triterpenes	In vitro, in vivo	↓ Glucose absorption + insulin resistance improves	[64]
*Amaryllidaceae*, *Asparagaceae*, *Asteraceae*, *Dioscoreaceae*, *Fabaceae*, *Liliaceae*, *Plantaginaceae*, *Smilacaceae*, *Solanaceae*, *Zygophyllaceae* families	Triterpenoid glycosides derived saponins	In vivo	↓ Blood glucose levels	[65]
*Aralia* spp.	Elatoside F, araloside C, chikusetsusaponin IVa, chikusetsusaponin IVa, elatoside I, oleanolic acid 3-O-*β*-D-glucopyranosyl(1 →3)-*α*-L-rhamnopyranosyl (1 → 2)-*α*-L-arabinopyranoside, kaurenoic acid, continental acid, 7-oxo-ent-pimara-8 (14), 15-dien-19-oic acid, 3-O-{β-D-glucopyranosyl-(1 → 2)-[*β*-D-glucopyranosyl -(1 → 3)]-*β*-D-glucuronpyranosyl} 28-O-*β*-D-glucopyranosyl ester of oleanolic acid, 3-O-{*β*-D-glucopyranosyl-(1 → 2)-[*β*-D-glucopyranosyl -(1 → 3)]-*β*-D-glucuronpyranosyl}olean-11,13(18)-diene-28- oic ester 28-O-*β*-D-glucopyranosyl	In vivo	↓ Blood glucose levels	[67]
*Cucurbitaceae* family	Cucurbitacin triterpenoids	In vitro, in vivo	↓ Blood glucose levels + insulin secretion stimulation	[69]
*Falcaria vulgaris* (Sickleweed)	α-pinene, spathulenol, carvacrol, limonene	In vitro, in vivo	↓ Blood glucose levels + insulin secretion stimulation + *β*-cells protection	[70]
*Urtica dioica* (Nettle)	4,7-Megastigma-diene-3, 9-diol; (3S,6R,7E,9R)-form, 3-ketone, 9-O-[ *β* -D-glucopyranosyl-(1⟶2)-b-d-glucopyranoside], 1-(3, 4-dihydroxyphenyl)-1, 2-propanediol; 3′-Me ether, (9Z,11E)-1, 3-hydroxy-9, 11-octadeca-dienoic acid, hexahydrofarnesyl acetone, geranyl acetone, (E)-anethole, *p*-hydroxybenzaldehyde, b-ionone	In vitro, in vivo	↓ Blood glucose levels + insulin resistance improves	[71]
*Polygonatum sibiricum* (Siberian Solomon’s Seal)	Curcumenol, geniposide	In vitro, in vivo	GSIS protein help and regulation, excess glucosa beta cell death protection	[85]
*Acanthopanax senticosus*	Triterpenoid saponins	In vivo	↓ Hepatic lipids and improve insulin resistance through AMPK activation	[123]

↓ reduce; ↑ activate.

### 2.7. Antimicrobial Effect

Finally, the effects on microbial growth are addressed. A total of twelve articles were found (Table 9).

Although microbial infection is not a cardiovascular disease itself, this section is addressed due to the role of pathogens in the development of endocarditis, a heart infection which can result in myocardial dysfunction and eventually heart failure [125]. It involves the nesting of microorganisms near the valves, gradually destroying them and rendering them useless. Although there are currently relatively few cases of endocarditis, studies related to this disease remain of interest due to its high mortality rate. It is worth noting that thanks to scientific advances, this disease has been successfully addressed, improving the prognosis of patients with this problem and preventing its onset [125].

Many of the compounds mentioned in the other sections have been found to have antimicrobial properties [56,78]. This feature would be useful as a secondary preventive measure against endocarditis while treating another heart condition.

Of all the plants, *Andrographis paniculata* is highlighted for its ability to protect against a wider range of pathogens [78].

**Table 9:** Studies demonstrating the antimicrobial effect.

Plant	Terpenoids	Study	Effect	Reference
*Coriander sativum* (Coriander), *Apium graveolens* (Celery), *Ocimum minimum* (Bush-basil)	Linalool, *α*-pinene, camphor, *p*-cymene	In vitro	*Ae. hydrophila*, *Ps. fragi*, *Ac. denitrificans*, *S. marcenscens*, *Sh. Putrefaciens*, *Y. lipolytica*, *S. cerevisiae*, *C. zeylanoides*, *D. hansenii*, *Pi. carsonii* inhibition	[53]
*Senna auriculata* (Avaram senna)	Oleanolic acid	In vitro, in vivo	*Escherichia coli*, *Salmonella typhi*, *Proteusmirabilis*, *Klebsiella pneumoniae*, *Bacillus subtilis*, *Staphylococcus aureus*, *Pseudomonas aeruginosa* inhibition	[56]
*Andrographis paniculata* (Creat)	Andrographatoside, oleanolic acid, andrographolide, 3-O-*β*-D-glucopyranosilandrographolide, 3-Oxo-14-deoxy-11,12-didehydroandrographolide, neoandrographolide, 14-deoxyandrographolide, andrograpanin, 3-O-*β*-D-glucosyl-14-deoxyandrographolide, 6′-acetylneoandrographolide, 14-deoxy-17-*β*-hydroxyandrographolide, 19-O-[*β*-D-apiofuranosyl(1 → 2)-*β*-D-glucopyranoyl]-3,14 dideoxyandrographolide, isoandrographolide, 14- deoxy-11-oxo-andrographolide, 14-deoxy-12-hydroxyandrographolide, 8,17-epoxy-14-deoxyandrographolide, 3-O-*β*-D-glucosyl-14-deoxyandrographolide, 12S-hydroxyandrographolide, 14-deoxy-15-isopropylidene-11,12-didehydroandrographolide, bisandrographolide A, bisandrographolide B, bisandrographolide C, bisandrographolide D, bisandrographolide E, bisandrographolide F, bisandrographolide G, bisandrographolide ether	In vivo	*Bacillus subtilis*, *Staphylococcus aureus*, *Escherichia coli*, *Pseudomonas aeruginosa*, *Bacillus anthracis*, *Micrococcus luteus*, *Staphylococcus epidermidis*, *Staphylococcus saprophyticus*, *Streptococcus pyogenes*, *Proteus mirabilis*, *Proteus vulgaris*, *Neisseria meningitidis*, *P. aeruginosa* inhibition	[78]
*Bougainvillea glabra* (Lesser bouginvillea)	Oleananoic acid acetate, oleoside dimethyl ester, goyaglucoside, oleanolic acid 3-O-beta-D-glucosiduronic acid, phytol, squalene, geranylgeraniol	In vitro	*Proteus vulgaris*, *Bacillus subtilis*, *Escherichia coli*, *Klebsiella pneumonia*, *Staphylococcus aureus* inhibition	[60]
*Houttuynia cordata* (Fish mint)	Houttuynin, decanal, trans-caryophyllene, decanoic acid, camphene, *β*-pinene, lauraldehyde, *α*-pinene, limonene, nonanol and linaloolbornyl acetate, methyl n-nonyl ketones, beta myrcene, monoterpene, 4-terpineol, caryophyllene oxide, derivatives phenylpropene, sesquiterpenes, oxidized diterpenes	In vivo	*Pseudomonas aeruginosa*, *E. coli* inhibition	[81]
*Pyrola* spp. (Pyrolae herba)	β-sitosterol, ursolic acid, uvaol, 3-hydroxy-11-oxo-oleanolic acid, 3,11-dioxo-oleanolic acid, monotropin, pisumionoside, daucosterol, pomolic acid, oleonolic acid, maslinic acid, collosic acid, taraxerol, miricadiol, betulin, ziyuglucoside I	In vitro	*Staphylococcus aureus*, *Klebsiella Pneumo-niae*, *Escherichia coli*, *Proteus vulgaris*, *Pseudomonas aeruginosa*, *Bacillus subtilis* inhibition	[63]
*Amaryllidaceae*, *Asparagaceae*, *Asteraceae*, *Dioscoreaceae*, *Fabaceae*, *Liliaceae*, *Plantaginaceae*, *Smilacaceae*, *Solanaceae*, *Zygophyllaceae* families	Triterpenoid glycosides derived saponins	In vivo	Gram-positive and Gram-negative inhibition	[65]
*Astragalus* spp, *Ginkgo biloba* (Ginkgo), *Panax pseudoginseng* (Notoginseng), *Phyllanthus emblica* (Emblic)	Astragaloside IV, diosgenin, ginsenoside Re, lupeol, oleanolic acid, phylloemblycin B, 20(S)-protopanaxtriol, ursolic acid	In vivo	Antimicrobial effect	[66]
*Aralia* spp.	Elatoside F, araloside C, chikusetsusaponin IVa, chikusetsusaponin IVa, elatoside I, oleanolic acid 3-O-*β*-D-glucopyranosyl(1 →3)-*α*-L-rhamnopyranosyl (1 → 2)-*α*-L-arabinopyranoside, kaurenoic acid, continental acid, 7-oxo-ent-pimara-8 (14), 15-dien-19-oic acid, 3-O-{*β*-D-glucopyranosyl-(1 → 2)-[*β*-D-glucopyranosyl-(1 → 3)]-*β*-D-glucuronpyranosyl} 28-O-*β*-D-glucopyranosyl ester of oleanolic acid, 3-O-{*β*-D-glucopyranosyl-(1 → 2)-[*β*-D-glucopyranosyl-(1 → 3)]-*β*-D-glucuronpyranosyl}olean-11,13(18)-diene-28-oic ester 28-O-*β*-D-glucopyranosyl	In vivo	*St. Aureus*, *E. coli*, *S. Dysenteriae* inhibition	[67]
*Isodon rubescens* (Donglingcao)	Oridonin, ponicidin, lushanrubescensin H, lushanrubescensin J, rhabdosin A, isodocarpine, rhabdoternin F, shikokianin, lasiodin, parvifolin AA, lasiodonin, lasiodoninacetonide, rostorin, isojiangrubesin C, isojiangrubesin E, rhabdoternin E, jaridonina, 14- O-acetyl-oridonin, isodonoiol, isodonal, rhabdosin B, efusanin A, xerophinoid B and 7,14-O-(1-methylethylidene) oridonin, ursolic acid, oleanic acid, β-sitosterol, α-amyrin, daucosterol, betulin, eryhrodiol, stigmasterol	In vivo	*Staphylococcus aureus*, *Streptococcus hemolyticus*, *E. coli*, *Staphylococcus albicans* inhibition	[68]
*Cucurbitaceae* family	Cucurbitacin triterpenoids	In vitro, in vivo	*E. coli*, *Bacillus cereus*, *Enterobacter faecalis*, *Salmonella paratyphi*, *Staphylococcus aureus*, *Proteus vulgaris* inhibition	[69]
*Zanthoxylum acanthopodium* (Andaliman)	Citronellol, geraniol, E-*β*-caryophyllene, 2-hexadecen-1-ol, ethyl linoleate, myrtenyl acetate, 5-(propenyl-2)-1,3,7-nonatriene, (E,E)-farnesylacetone, farnesol, citronellyl propionate, geranyl hexanoate, citronellyl acetate, 1,5,9-decatriene,2,3,5,8-tetramethyl, neryl butanoate	In vitro	*S. aureus*, *S. typhimurium* y *Mycobacterium smegmatis* inhibition	[126]

### 2.8. Practical Uses

**Figure 4:**
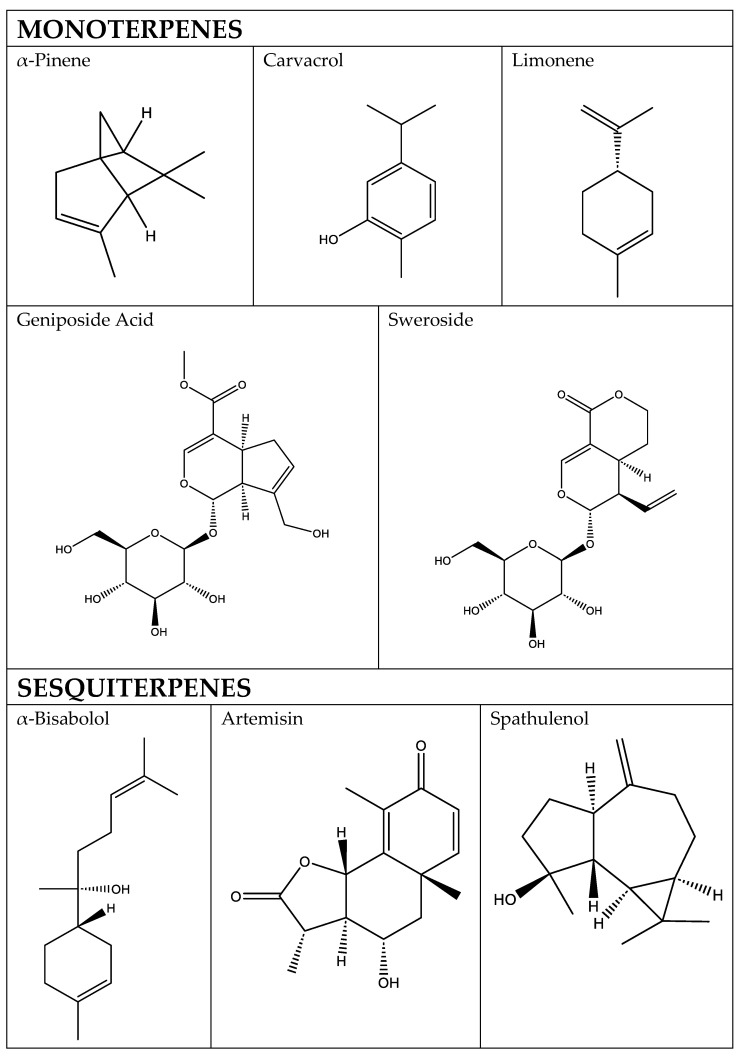

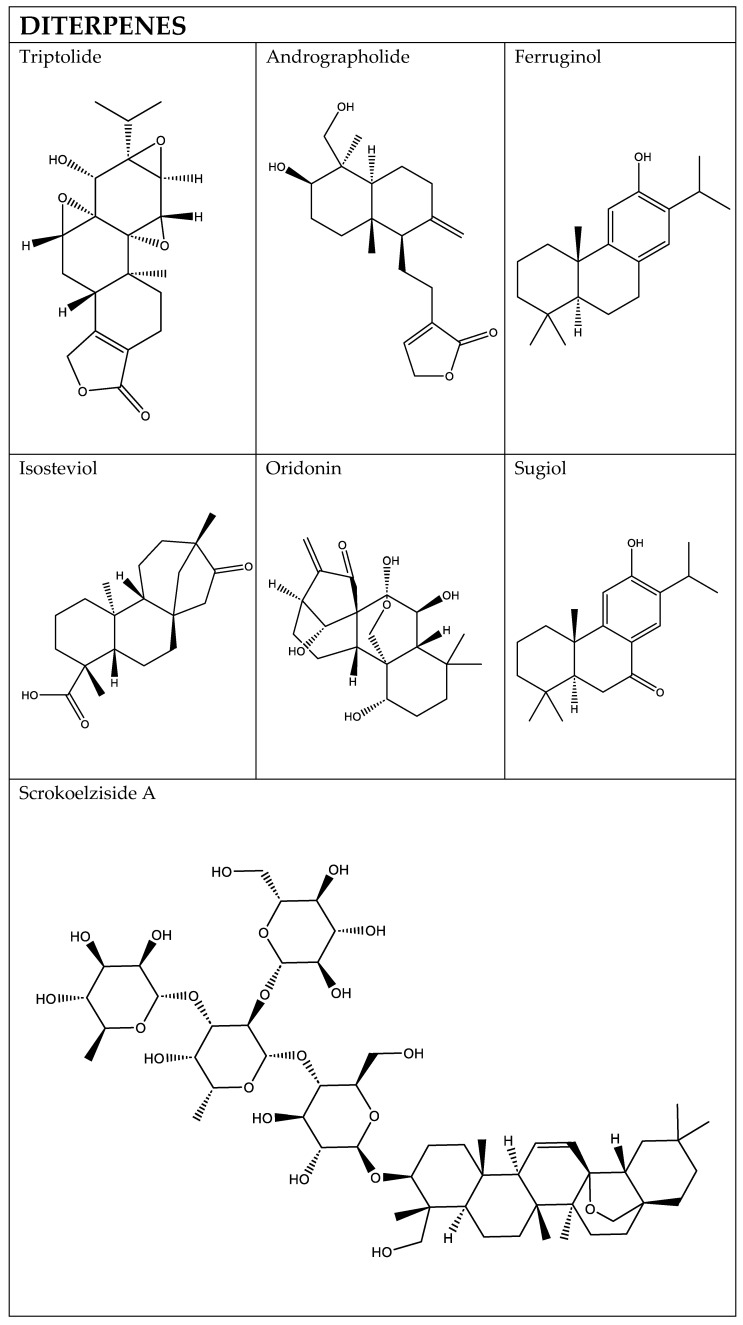

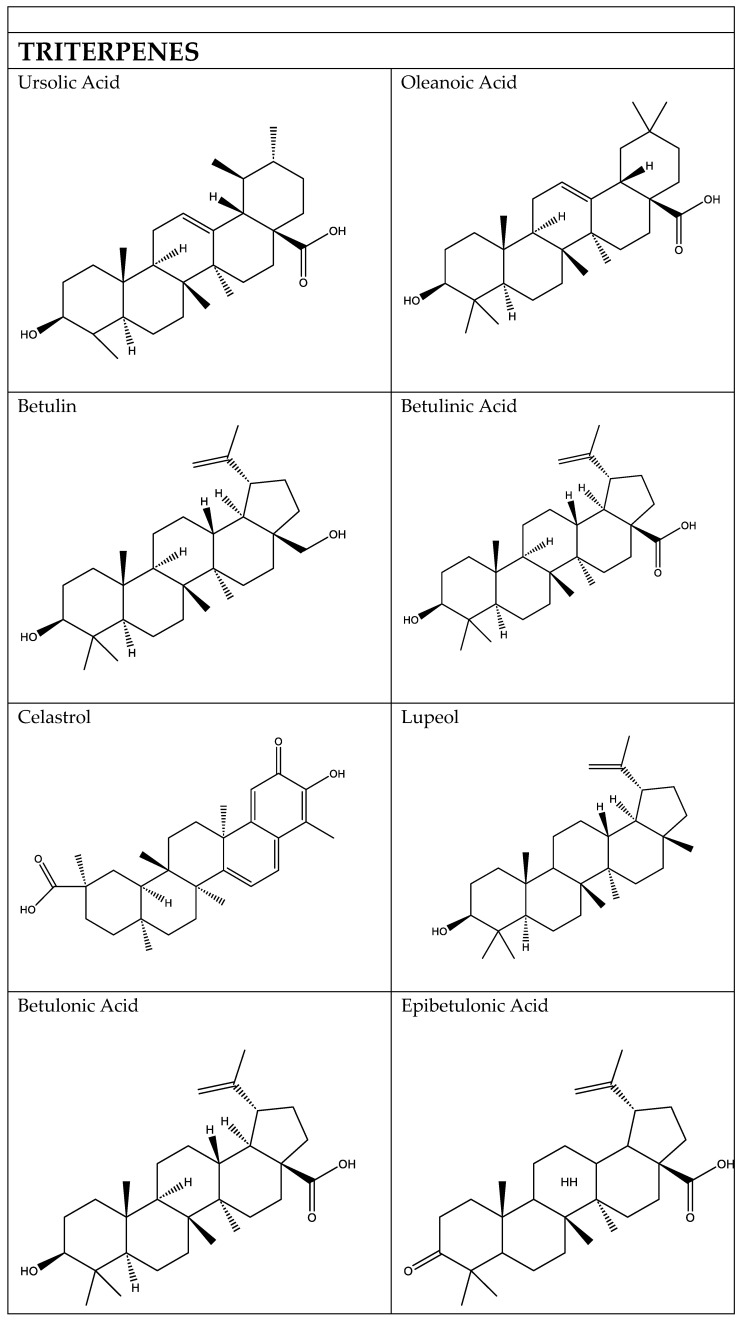

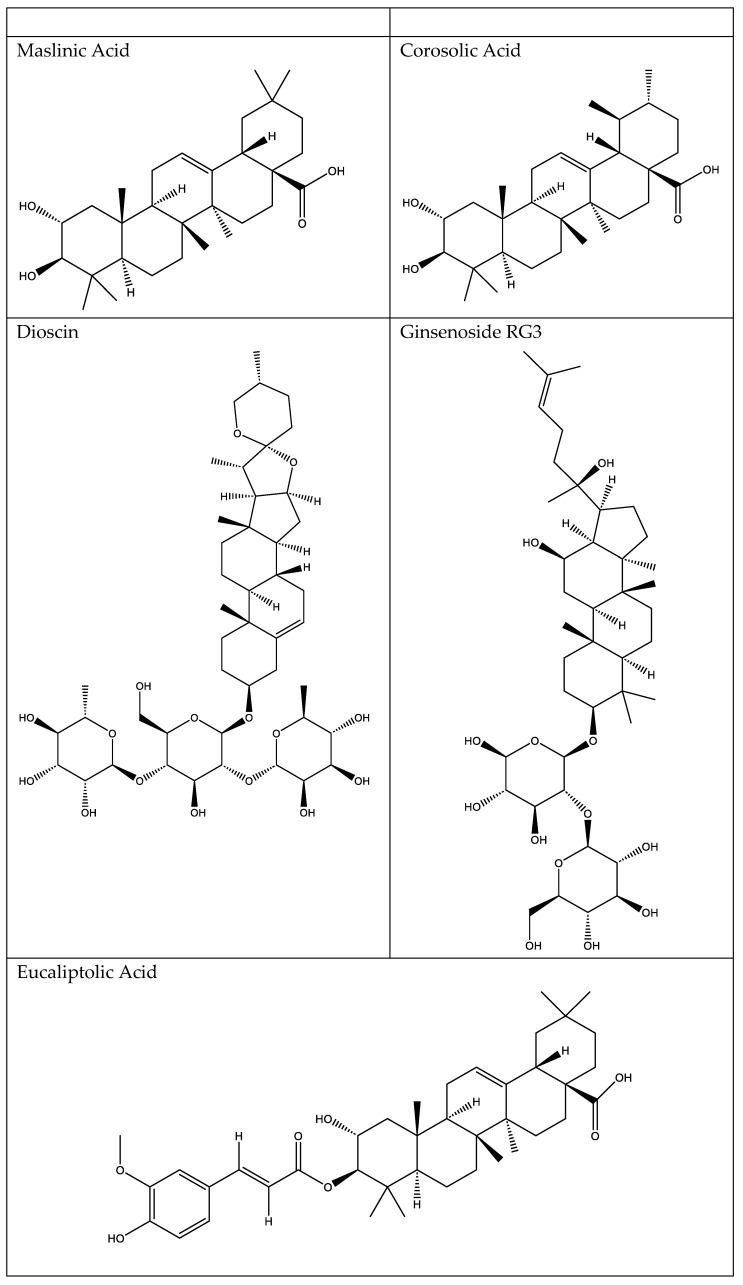
Chemical structure of terpenes with cardioprotective properties.

This section focuses on the application of the previously described properties in the prevention and treatment of various heart diseases. The selected diseases are myocardial ischemia, heart failure, arrhythmia, and hypertension. These diseases were chosen because they are the most frequently addressed in literature. Figure 4 illustrates the chemical structure of the most frequently reported terpenes with healing properties, classified by chemical family.

#### 2.8.1. Myocardial Ischemia

Myocardial ischemia occurs due to the reduction or interruption of blood flow through the coronary arteries that supply the heart [127]. This obstruction typically results from the blockage of these arteries, mainly due to cholesterol plaques formed within the arterial walls [112]. The interruption of blood flow and oxygen deprivation affects the energy load of the myocardium by gradually reducing ATP production until consumption exceeds production, causing cell death [127]. Once blood flow is restored, the necrotic portion of the heart begins to deform due to the contractions and internal pressure of the ventricles and atria. This deformation reduces the organ’s performance and increases the possibility of rupture through the necrotic tissue, causing more serious problems [93].

In this situation, terpenoids may be effective thanks to their antioxidant (Isosteviol, lupeol), anti-inflammatory (α-Bisabolol, Sugiol, Lupeol, Ursolic acid, Oleanonic acid, Eucalyptolic acid, Scrokoelziside A, Scrokoelziside B, 14-deoxyandrographolide), anti-apoptotic/pyroptotic (α-Bisabolol and Ferruginol), anti-remodeling (Artemisin, Betulin, Celastrol, Dioscin, Geniposide, Ginsenoside Rg3, Oridonin, Sweroside, Triptolide), antiatherosclerosis (Oleanolic acid, betulinic acid, corosolic acid, maslinic acid, epibetulinic acid and betulonic acid) and antidiabetic (oleanolic acid, α-pinene, spathulenol, carvacrol, limonene) properties. The case of the triterpene lupeol acetate from methanolic extract of *Cleome viscosa* leaves supposes a promising therapeutic option for MI due to its favorable pharmacokinetics and safety profile [90].

**Figure 5:**
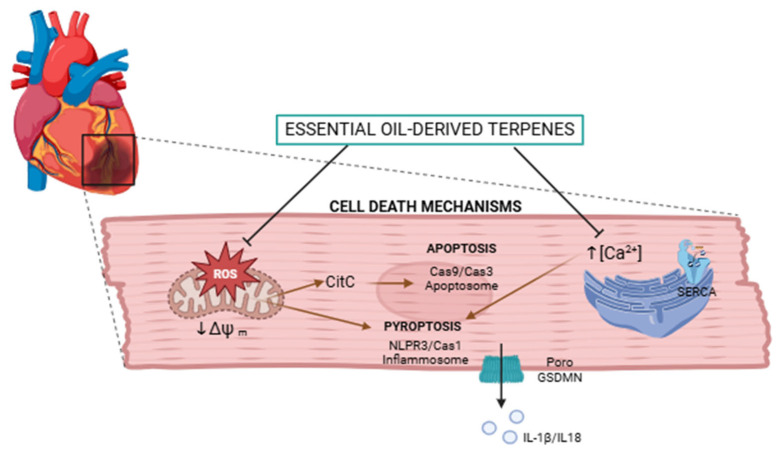
Molecular mechanisms of cell death in MI modulated by essential oil-derived terpenes. MI occurs when blood flow to a region of the heart is reduced or completely blocked, preventing oxygen from reaching the tissue and leading to cardiomyocyte death. The main cell death pathways involved are apoptosis and pyropotosis; both are targeted by highly interrelated molecular alterations which include mitochondrial dysfunction (∆*Ψm*), ROS burst and increase intracellular Ca^2+^ levels. Figure made with BioRender.

Through its antioxidant effect, the reduction of ROS levels in cardiomyocytes promotes optimal cellular and mitochondrial function. This delays cell death and allows more time for the restoration of blood flow [28]. The anti-inflammatory effect mitigates the inflammatory response to cell death and inhibits many of the inflammatory compounds released into the intercellular space following cardiomyocyte death [72]. The reduction in inflammatory molecule level in the interstitial tissue prevents the onset of pyroptosis [16] (Figure 5).

Besides apoptotic and pyroptotic cell death, another cell death process responsible for MI injury is ferroptosis, triggered by an increase in lipid peroxidation level and accumulation of ferrous ion [128]. Plant-derived triterpenes are a good approach to prevent it and reduce myocardial damage. In this line, a recent study has proven the anti-ferroptotic effect of the triterpenes dioscin and ginsenoside Rg3 in MI [129,130].

Once blood flow is restored, the priority is to minimize the adverse effects of tissue necrosis. In this context, the anti-remodeling property may help reduce the distension of the resulting scar tissue and maintain the stability of both the tissue and the cells attached to it, thereby preventing the risk of rupture of the heart wall [16,78].

Finally, as a preventive measure against coronary artery blockage, the anti-atherosclerotic and antidiabetic properties of certain compounds may contribute to the prevention of this type of heart disease. The anti-atherosclerotic effect can reduce the deposition of cholesterol, in the form of plaques, within the arteries, thereby preventing their obstruction [24,55,65]. The antidiabetic property can help regulate blood glucose and cholesterol levels, decreasing the possibility of them clumping in the arteries [24,55].

#### 2.8.2. Heart Failure

Heart failure occurs when the heart is unable to function properly, resulting in weaker contractions and reduced blood flow. This condition often develops as a consequence of other heart diseases, primarily ischemia and hypertension. Myocardial weakness arises from the stress induced by these conditions and the loss of cardiomyocytes, gradually diminishing myocardial performance and contractile strength [131].

In this context, the diverse effects of terpenoids in maintaining cardiomyocyte health could slow down and even reverse the harmful consequences of heart failure. The most relevant properties are those involved in cellular protection and maintenance, including antioxidant (isosteviol), anti-inflammatory (α-bisabolol, sugiol, lupeol, ursolic acid, oleanonic acid, eucalyptolic acid, scrokoelziside A, scrokoelziside B, 14-deoxyandrographolide, corosolic acid), anti-apoptotic (α-bisabolol and ferruginol) and anti-remodeling (artemisinin, betulin, celastrol, dioscin, geniposide, ginsenoside Rg3, oridonin, sweroside, triptolide).

Thanks to their antioxidant properties, terpenoids help to maintain low levels of ROS. The heart, a continuously active organ, is known to produce very high amounts of ROS due to its constant energy demand [27]. Maintaining low ROS levels supports the proper functioning of the cellular machinery and prevents the activation of apoptotic signaling pathways [25]. This control of ROS levels can be achieved through terpenoids that help neutralize ROS [58,66,69] and activate the enzymes responsible for their neutralization [26,57,64].

The anti-inflammatory function, responsible for reducing the inflammatory response, may prevent an uncontrolled response and the activation of pyroptosis. Furthermore, it also limits the accumulation of ROS, which could otherwise impair cellular function [72]. Terpenoids regulate the inflammatory response by reducing the levels of inflammatory molecules [66,68]. In addition, their interactions with immune system components help modulate and limit the inflammatory response [70,79].

Finally, to increase the number of cardiomyocytes and slow down the natural process of apoptosis, terpenoids have been found to integrate into cell signaling processes and redirect all the routes that activate the apoptosis process [24,26,55].

#### 2.8.3. Arrhythmia

Arrhythmia is a heart condition that disrupts the regularity of the heartbeat and the synchronous contractions of the atria and ventricles. It can arise from various causes, including mutations in the ion channels responsible for the action potential, structural malformations of the heart, or external substances that interfere with the normal function of the heart’s endogenous pacemakers [99]. Thanks to the wide variety of terpenoids (*Eleutherococcus lupane* triterpenes) and their diverse properties, they can be used to treat arrhythmia depending on the underlying cause. They primarily achieve this effect by interacting with ion channels that regulate and propagate the action potential throughout the heart. Terpenoids can be categorized into three main groups, depending on their mechanism of action on cardiomyocyte electrophysiology: those that control and regulate calcium channels involved in generating the plateau phase of the action potential [15,101]; those that interact with and regulate the sodium and potassium channels, responsible for the depolarization and repolarization phases, respectively [55,103]; and those that protect against substances that disrupt the proper functioning of the cardiac nodes [24,64,69].

**Figure 6:**
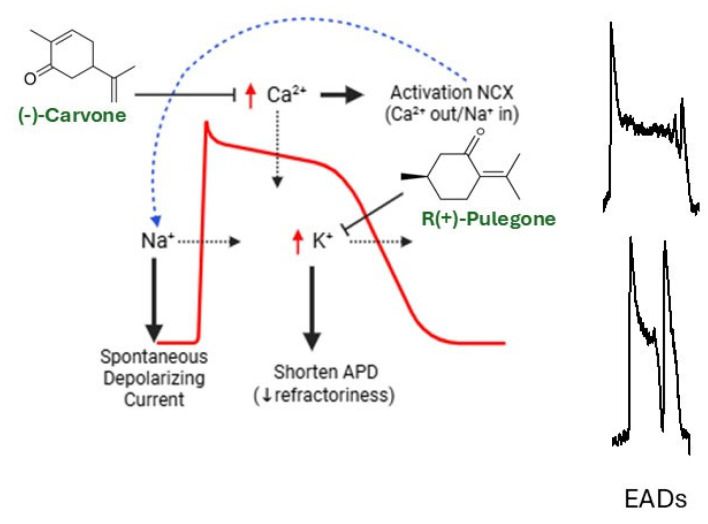
Electrophysiologica mechanisms underlying arrhythmic events in cardiomyocytes and terpene candidates proposed to counteract these proarrhythmic pathways. Figure made with BioRender.

Figure 6 shows a schematic and simplistic view of the cardiac action potential with the depolarizing Na^+^ current, plateau Ca^2+^ current and repolarizing K^+^ channel. The dysregulation of potential action currents is responsible for cardiac arrhythmia. For example, in atrial fibrillation (AF), multiple electrophysiological disturbances contribute to the initiation and maintenance of the arrhythmia. Enhanced sympathetic tone increases sinoatrial node automaticity through upregulation of L-type calcium channel activity, leading to intracellular Ca^2+^ overload. To compensate the high level of cytosolic Ca^2+^ the Na^+^/Ca^2+^ exchanger (NCX) is overactivated, resulting in elevated cytosolic Na^+^ levels. Concurrently, augmented repolarizing K^+^ currents shortens the action potential duration (APD), reducing cellular refractoriness. The combination of increased intracellular Na^+^ and abbreviated refractoriness promotes proarrhythmic events such as early and delayed afterdepolarizations (EADs and DADs), which can trigger arrhythmic events as AF [132]. Two monoterpenes, (-)-carvone and R(+)-pulegone, have shown the ability to block Ca^2+^ and K^+^ currents, respectively; it could convert both compounds into good candidates to treat arrhythmic events such as AF.

#### 2.8.4. Hypertension

The narrowing of blood vessels due to the contraction of their smooth muscles increases the internal pressure generated by blood flow, leading to hypertension and causing various disorders throughout the body [106]. This condition can also induce cardiac remodeling, particularly in the ventricles, reducing the efficiency of each contraction and potentially leading to heart failure [133].

As with arrhythmia, the wide variety of terpenoid compounds and their diverse properties (*α*-bisabolol, oleanolic acid, betulinic acid, corosolic acid, maslinic acid, epibetulinic acid and betulonic acid) allow hypertension to be addressed through multiple mechanisms. One group of terpenoids interacts with calcium channels that regulate the contraction of arterial smooth muscle [15,59,83], while another group inhibits the signaling pathways that trigger vessel contraction [26,64,70].

### 2.9. Perspective and Future Research

Future research should focus on addressing the current gaps limiting the clinical translation of basic research on natural compounds with cardioprotective activity, such as essential oils and terpenoids. Further studies are needed to elucidate the precise molecular mechanisms underlying their cardioprotective effects, preferably using human cardiomyocytes models, since most available evidence comes from experimental animal models due to the limited accessibility of human cardiac tissue. This limitation can be overcome by employing human induced pluripotent stem cell derived cardiomyocytes, which not only resolve the ethical concerns associated with animal experimentation but also provide a more reliable and physiologically relevant human in vitro model.

Another weak point of terpenoids as therapeutic agents lies in their very low yields in plants (often below 0.05% of dry weight), combined with prolonged growth cycles (e.g., Panax ginseng requires 5–7 years to reach maturation). In addition, the biochemical profile and composition of these plants can vary due to environmental conditions. Altogether, their extraction from native plants is neither scalable nor economically sustainable [134]. The main solution to this issue is to pass extracts throughout chemical synthesis, a method that is not cost-effective due to their molecular complexity which manifests in high production cost [135]. These limitations justify the development of alternative production methods, such as heterologous biosynthesis in optimized microbial or plant chassis. Metabolic engineering of native plants, microbial terpenoid production and heterologous plants have benefitted from technologies such as CRISPR-Cas9 [134].

Additionally, another milestone to address involves the development of innovative delivery systems aimed at overcoming the limited bioavailability of these molecules, a limitation that restricts their potential as effective pharmacological approaches. The low bioavailability is mainly attributed to its low stability and poor solubility. To get over this limitation, scientists’ efforts have focused on designing strategies to enhance the bioavailability of terpenoids [136].

One approach involves the use of micro- and nanoencapsulated essential oils and terpenes, which protect these compounds from oxidation, prevent undesirable interaction with other molecules, and enable controlled release. The last point is very important, as it enhances their release in the gastrointestinal tract and consequently improves absorption [137,138,139,140].

The particles containing the natural compound are referred to as microparticles, consisting of an encapsulated material (core) and the encapsulating agent, which may include natural biopolymers of proteins, carbohydrates and lipids as well as synthetic ones. Depending on the encapsulating technique, two structures can be obtained: “microcapsules”, where the core is concentrated in the center coated by a continuous “wall” of encapsulating agent, and “microspheres”, where the active compound is dispersed throughout the matrix, which implies the exposure of the core on the surface. A key requirement for this technological approach is the selection of encapsulating agents that are chemically inert and compatible with the encapsulated compound. The release of the encapsulated agent can be triggered by different conditions such as temperature and pH [137,138].

Among nanotechnology-based delivery platforms, liposomes exhibit remarkable potential to improve the bioavailability of natural compounds used as therapeutic agents. Their nanoscale dimensions and surface characteristics enhance the bioavailability of the compound carried through facilitating lymphatic absorption and enhancing cellular uptake and controlled release. These features contribute to prolonged circulation time and the possibility to accumulate them in the target tissue due to the coupling with antibodies or adaptamers [139,140]. To summarize, the above-mentioned encapsulated systems improve the pharmacokinetic profiles of natural compounds and reduce systemic side effects.

Nonetheless, plant-derived bioactive molecules are valuable templates for medicinal chemistry and offer structurally rich scaffolds that can be optimized to generate more potent, stable, and clinically viable therapeutic agents.

## 3. Conclusions

As has been demonstrated, the terpenoid family comprises a large and diverse family of natural compounds with countless medicinal properties. Recent evidence has shown that the pharmacological potential of certain plants traditionally used in medicine is largely due to their high terpene content. The terpenoids with cardioprotective effects identified to date come from a remarkable biodiversity of plant sources, ranging from genera such as *Aralia*, *Astragalus*, *Betula*, and *Hypterygium*, and families such as Amaryllidaceae and Cucurbitaceae, to species such as *Liquidambar orientalis* and *Ginkgo biloba*. This diversity, together with the positive results reported in experimental studies, highlights the vast array of therapeutic possibilities offered by these compounds.

Among the wide variety of biological actions attributed to terpenoids, their antioxidant and anti-inflammatory effects stand out as particularly relevant for cardiovascular protection, a field which constitutes a big challenge to researchers and clinicians due to its high global burden. It is also noteworthy that many plants, such as the *Aralia* genus and *Swertia chirayita*, are present across multiple functional categories, making them versatile candidates for the development of treatments targeting different cardiovascular disorders.

Overall, ongoing research on both well-characterized and newly discovered terpenoids is expected to reveal novel strategies for addressing cardiac pathologies, potentially offering therapeutic benefits in ways that current medications cannot achieve, with the additional advantage of their generally low toxic profile. This review aims to support and guide future research into this family of chemical compounds, emphasizing the need to address the current gaps that limit the clinical translation of experimental results. One of the main limitations is the scarcity of available data employing in vitro human models, an issue that could be overcome using human induced pluripotent stem cells derived from cardiomyocytes. In addition, their low bioavailability and the limited yield to obtain them from natural sources are obstacles to be considered in their therapeutic development. These limitations highlight the need for further research into advanced delivery systems and biotechnological production strategies capable of improving both their pharmacokinetic profile and large-scale availability.

## Data Availability

Further inquiries can be directed to the corresponding author(s).

## Abbreviations

The following abbreviations are used in this manuscript:

AC	Antioxidative Capacity
ACE	Angiotensin Converting Enzyme
AMPK	AMP-activated Protein Kinase
ATP	Adenosine Triphosphate
AV	Atrioventricular Node
CAT	Catalase
Col I	Collagen Type I
Col III	Collagen Type III
COX2	Prostaglandin-Endoperoxide Synthase
DMAP	4-Dimethylaminopyridine
DMAPP	Dimethylallyl diphosphate
GPx	Glutathione Peroxidase
GSH	Reduced Glutathione Tripeptide
GST	Glutathione S-Transferase
HDL	High-Density Lipoprotein
HMGR	3-Hydroxy-3-methylglutaryl-CoA reductase
IFN-ɣ	Interferon ɣ
IL-1	Interleukin 1
IL-1β	Interleukin 1β
IL-6	Interleukin 6
IL-8	Interleukin 8
IL-10	Interleukin 10
IL-12	Interleukin 12
iNOS	Nitric Oxide Synthase
IPP	Isopentenyl Pyrophosphate
JNK	c-Jun N-terminal Kinase
LDL	Low-Density Lipoprotein
MMP-9	Matrix Metallopeptidase 9
NADPH	Nicotinamide Adenine Dinucleotide Phosphate
NO	Nitric Oxide
PGE2	Dinoprostone
PGI2	Prostacyclin
ROS	Reactive Oxygen Species
SOD	Superoxide Dismutase
TGs	Triglycerides
TNF-α	Tumor Necrosis Factor α
VLDL	Very Low-Density Lipoprotein
